# Architecture of The Human Ape1 Interactome Defines Novel Cancers Signatures

**DOI:** 10.1038/s41598-019-56981-z

**Published:** 2020-01-08

**Authors:** Dilara Ayyildiz, Giulia Antoniali, Chiara D’Ambrosio, Giovanna Mangiapane, Emiliano Dalla, Andrea Scaloni, Gianluca Tell, Silvano Piazza

**Affiliations:** 1 Laboratory of Molecular Biology and DNA repair, Department of Medicine, University of Udine, p.le M. Kolbe 4, 33100 Udine, Italy; 2 Proteomics and Mass Spectrometry Laboratory, Institute for the Animal Production System in the Mediterranean Environment (ISPAAM), National Research Council (CNR) of Italy, via Argine 1085, 80147 Naples, Italy; 3 Bioinformatics Core Facility, Centre for Integrative Biology (CIBIO), University of Trento, via Sommarive 18, 38123 Povo (Trento), Italy

**Keywords:** Cancer genomics, Cancer genomics, Gene regulatory networks, Gene regulatory networks

## Abstract

APE1 is essential in cancer cells due to its central role in the Base Excision Repair pathway of DNA lesions and in the transcriptional regulation of genes involved in tumor progression/chemoresistance. Indeed, APE1 overexpression correlates with chemoresistance in more aggressive cancers, and APE1 protein-protein interactions (PPIs) specifically modulate different protein functions in cancer cells. Although important, a detailed investigation on the nature and function of protein interactors regulating APE1 role in tumor progression and chemoresistance is still lacking. The present work was aimed at analyzing the APE1-PPI network with the goal of defining bad prognosis signatures through systematic bioinformatics analysis. By using a well-characterized HeLa cell model stably expressing a flagged APE1 form, which was subjected to extensive proteomics analyses for immunocaptured complexes from different subcellular compartments, we here demonstrate that APE1 is a central hub connecting different subnetworks largely composed of proteins belonging to cancer-associated communities and/or involved in RNA- and DNA-metabolism. When we performed survival analysis in real cancer datasets, we observed that more than 80% of these APE1-PPI network elements is associated with bad prognosis. Our findings, which are hypothesis generating, strongly support the possibility to infer APE1-interactomic signatures associated with bad prognosis of different cancers; they will be of general interest for the future definition of novel predictive disease biomarkers. Future studies will be needed to assess the function of APE1 in the protein complexes we discovered. Data are available via ProteomeXchange with identifier PXD013368.

## Introduction

Alteration of DNA repair mechanisms is an important hallmark of cancer cells, and plays a role both in the onset of an initial cancerous phenotype and in tumor progression. Tumor cells can develop drug resistance through repair mechanisms that counteract the DNA damage induced by chemotherapy or radiotherapy^1,2^. Thus, specific DNA repair inhibitors are often combined with DNA-damaging agents to improve therapy efficacy. Emerging evidences in tumor biology suggest that: i) protein-protein interactions (PPIs) specifically modulate both canonical and non-canonical roles of DNA repair enzymes; ii) RNA processing pathways participate in DNA-Damage Response (DDR); iii) defects in the above-mentioned regulatory mechanisms are associated with cancer genomic instability^3^. Very recent studies clearly show that many DNA repair proteins are associated with those involved in RNA metabolism, proving a role of their interactome network in undertaking non-canonical functions affecting gene expression in tumors. In addition, novel studies have shown that interaction of DDR components and miRNA biogenesis process is linked to cancer development^2^. In the context of these emerging lines, we already demonstrated the crucial role that enzymes belonging to the base excision DNA repair (BER) pathway play^4^. In particular, we showed that the essential BER enzyme apurinic/apyrimidinic endonuclease 1 (APE1), which is encoded by the *APEX1* gene, contributes to the regulation of oxidative stress responses and to the expression of chemoresistance genes *via* unsuspected functions in RNA metabolism^4–8^. The involvement of this protein in RNA processing events^9–11^, including miRNA expression, was recently unraveled by our group using a limited unbiased functional proteomic approach^4^. However, the reduced characterization of APE1 interaction with proteins involved in miRNA processing, *e.g*. NPM1, hnRNPU, PRP19, SFPQ and p53^12^, strongly limited our complete understanding of this phenomenon and of its functional relevance in cancer biology, thus hampering a further translation of these findings into therapy.

It is well known that the different functions of APE1 may depend on its interacting partners^13,14^; for example, an alteration of its interaction networks has been reported to play a significant role in BER impairment. A recent hypothesis also suggests that APE1 functional dysregulation may impact on the RNome expression and, thus, on the expression of target genes playing a relevant role in the pathology onset^4^. Since several cancer-associated APE1 variants present mutations in the endonuclease domain, exhibiting only mild nuclease defects *in vitro*^15–17^, we hypothesize that APE1 non-canonical functions associated with its overexpression and/or an altered expression of its protein interacting partners should be related to cancer promotion. A proof of concept for the relevance of APE1 PPIs in cancer biology is represented by the paradigmatic example of the APE1-nucleophosmin 1 (NPM1) interaction, as ascertained by us^18^. NPM1 is a multifunctional protein that controls cell growth and genome stability through a mechanism either involving nucleolar-cytoplasmic shuttling or a fine modulation of the whole BER pathway^11,19^. Recently, an important role for NPM1 in miRNA biology, associated with cancer development, was outlined^20–22^. Abnormal cytoplasmic APE1 and NPM1 levels were associated with the oncogenic progression and chemoresistance of HGSC (high-grade serous ovarian adenocarcinoma), a prediction of a poor prognosis therein^23,24^, and a dysregulation of the BER and miR-221/222 processing pathways in AML cells^18,25^. The efficacy of novel APE1/NPM1 interaction inhibitors, which sensitize cancer cells to chemotherapy agents, supports the translational importance of these findings^26^. These results further support the hypothesis that an alteration of other APE1 PPIs may be causally involved in cancer development and chemoresistance.

Prompted by these observations, the present work was aimed at: i) implementing the already known APE1-PPI network using a more efficient functional proteomics approach, and ii) defining the association of the APE1-PPI network with the modulation of tumor progression and chemoresistance through a systematic bioinformatics analysis of the Cancer Genome Atlas (TCGA) datasets. To this purpose, we used HeLa cells as a general and well-characterized cancer cell model to generate novel disease hypotheses and molecular diagrams. Having a well-characterized cell model is essential when using unbiased strategies, such as genomics and proteomics, in order to easily interpret data and generate novel assumptions. In this context, few years ago, we developed a specific HeLa cell line stably expressing a flagged tagged APE1 form for selective and efficient protein complex immunocapture^4,17,26,27^. This cell line, and its corresponding products, were characterized in both cancer and genotoxic damage response contexts by means of different holistic approaches, including genomics, transcriptomics and proteomics^4,12^. In this study, we wanted to improve our previous interactomic analyses by taking advantage of more sensitive mass spectrometry technologies, which were here applied to the analysis of extracts from different subcellular compartments. Resulting data were analysed by bioinformatics in a dedicated translational perspective. Our findings support the possibility to infer APE1 interactomic signatures associated with bad prognosis of different cancers and will be of general interest for the definition of novel predictive biomarker signatures of cancers.

## Results

### Proteomic characterization of the APE1-PPI network

**Figure 1:**
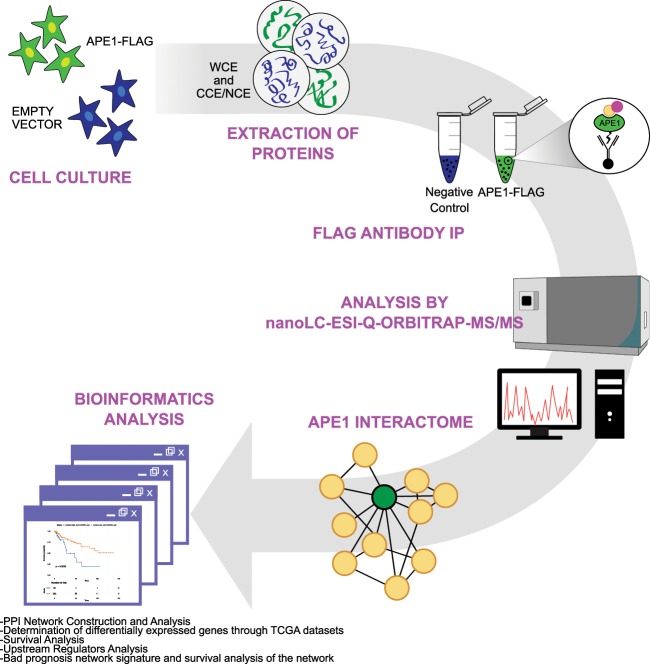
Schematic experimental pipeline used in this study. Schematic representation of the workflow for ascertaining APE1-interacting proteins by proteomic experiments. Whole-cell lysates (WCE), or nuclear (NCE) and cytoplasmatic (CCE) extracts were prepared from the 3 × APE1 FLAG-tagged expressing cells or control cells transformed with an empty vector (SCR). M2 antibodies against the FLAG peptide were used for co-immunoprecipitation of the above cell lysates. As additional control experiment, identical cell extracts from HeLa cells expressing 3 x APE1 FLAG-tagged were also co-immunoprecipitated with a resin lacking the FLAG antibody (res). The resulting bound proteins were digested with trypsin and analyzed by nanoLC-ESI-Q-Orbitrap-MS/MS. By comparison with proteins identified in the control co-immunoprecipitation experiments, we removed contaminant proteins from components identified in WCE, NCE and CCE; the resulting APE1-interacting partners were further added of additional proteins binding to APE1 (n = 80) from previous studies^4^, and finally subjected to bioinformatics analysis.

**Figure 2:**
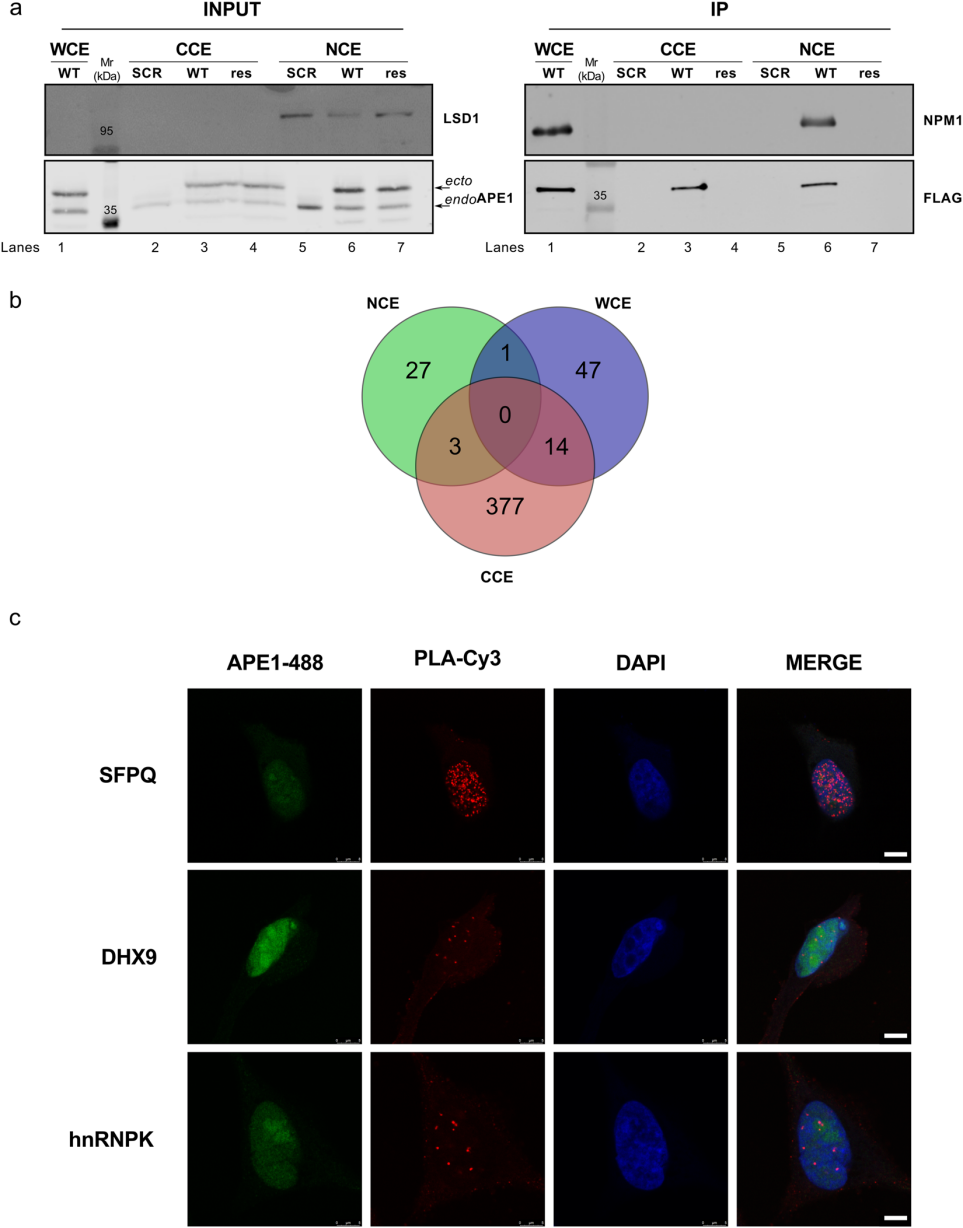
Proteomic characterization of the APE1 interactome. (**A**) Representative Western blotting to confirm APE1 pulldown in the co-immunoprecipitation experiment. Western blotting analysis was performed on total HeLa cell clone extracts (INPUT) and on co-immunoprecipitated material (IP) with specific antibodies for APE1 and FLAG. The endogenous (*endo*) and ectopic (*ecto*) form of the APE1 protein is visible. The resulting material was tested for the occurrence of NPM1, a known APE1 interactor. LSD1 was used to probe nuclear enrichment. SCR, HeLa cell clone transfected with empty vector; WT, HeLa cell clone expressing APE1-FLAG tagged protein; res, co-immunoprecipitated with a resin lacking the FLAG antibody; WCE, whole cell extract; NCE, nuclear cell extract; CCE, cytoplasmic cell extract. (**B**) Venn diagram showing APE1-interacting partners identified in whole-cell lysates (WCE), or nuclear (NCE) and cytoplasmatic (CCE) extracts. (**C**) Nucleoplasmic interaction between APE1 and three identified interactors. HeLa cells were seeded on a glass coverslip and the PLA reaction was carried out using anti-APE1, anti-SFPQ, anti-DHX9 and anti-hnRNPK antibodies. APE1 localization was detected by using an anti-APE1 antibody and visualized in green. Confocal microscopy analysis highlighted the presence of distinct fluorescent red dots (PLA signals) indicating the occurrence of *in vivo* interaction between APE1 and its protein partners. DAPI staining was used as a reference for the nuclei. See also Supplementary Figs. S1 and S2 for negative controls. Bars, 8 µM.

With this work, we wanted to study the relevance of APE1-PPIs in cancer using an unbiased functional proteomic approach in order to expand the number of known APE1 PPIs, as derived from studies from this and other groups^4,12^. To this purpose, we used HeLa cells stably expressing the APE1 FLAG-tagged protein^12^ (WT), which were here managed to optimize the isolation of APE1-PPI complexes through co-immunoprecipitation experiments (Figs. 1 and 2A). Differently from our previous investigations^12^, APE1-interacting protein complexes were isolated from either the whole cell lysate, or nuclear- and cytoplasmic-enriched subcellular fractions. Then, resulting protein mixtures were resolved by SDS-PAGE, and corresponding gel lanes were cut into parallel gel portions that were further subjected to proteomic analysis. As control experiments, we applied the same proteomic procedure to a cell clone stably transfected with the empty scramble vector (SCR) (Figs. 1 and 2A). As additional negative control experiments, nuclear and cytoplasmic cell extracts from HeLa cells expressing APE1 FLAG-tagged were co-immunoprecipitated with a resin lacking the anti-FLAG antibody to exclude any additional background (Fig. 2A, res). Western blotting analysis showed an APE1 enrichment in co-immunoprecipitated material from nuclear, cytoplasmic and total cell extracts of a HeLa cell clone stably expressing the flagged protein (Fig. 2A). In order to check for the quality of the immunoprecipitated materials, nucleophosmin 1 (NPM1) was used as a known APE1 interactor prior to further proteomic analysis^12,18,19,28^. By using this approach, a number of proteins were identified in APE1-FLAG co-immunoprecipitates from the above-mentioned whole-cell lysate and corresponding subcellular fractions (Supplementary Table S1 and Table S2). After careful filtration for false positives identified in the corresponding control (SCR and res) samples, 62, 31 and 394 proteins were identified as potential APE1 interactors in the whole-cell lysate, nuclear fraction and cytoplasmic fractions, respectively, which accounted for 455 non-redundant proteins. A poor overlapping of components from different extracts was observed, qualitatively confirming the preparation specificity (Fig. 2B). Various molecules (*i.e*. FEN1, hnRNPK, NPM1, PABPC1, SFPQ and XRCC1) were already described as APE1-interacting partners in other studies from this^4,12^ and other groups^24,29,30^, confirming the good quality of our analysis. The following proximity ligation assays were then successfully carried out to validate the identified APE1-interacting proteins within cells: i) SFPQ (splicing factor, proline- and glutamine-rich), DHX9 (DEAH-box helicase 9) and hnRNPK (heterogeneous nuclear ribonucleoprotein K) in HeLa cells (Fig. 2C and Supplementary Figs. S1 and S2); ii) hnRNPA2/B1 in the JHH-6 hepatocellular carcinoma cell line^31^ (Supplementary Fig. S1B); iii) SFPQ in the A549 lung cancer cell line (Supplementary Fig. S1C)^32^. The above-mentioned APE1-binding partners list was then added with additional proteins (n = 80) deriving from previous APE1-focused interactomic investigations^4^ to yield a final list of APE1-PPI elements (n = 535), which were associated with 531 non-redundant genes. The large size of this inventory (containing direct as well as indirect protein-binding partners) was rationalized based on the multiple biological functions/activities in which APE1 has been associated (redox control, transcriptional activity, DNA and RNA metabolism), and its localization in various subcellular districts. This condition is similar to the one recently reported for two APE1 protein interactors, namely XRCC6 and XRCC5, which have been similarly demonstrated to bind both to RNA/DNA as well as to about 300 proteins^33^. Other examples of proteins having hundreds of interactors (as deduced by a single immunocapture experiment) are already present in the scientific literature^34–37^. On the other hand, this final list, of direct or indirect APE1-binding partners, contained about 100 proteins whose capability to interact (directly or indirectly) with APE1 was already ascertained by different experimental approaches (Supplementary Table S3). The above-mentioned protein inventory was then subjected to bioinformatics analysis to establish a PPI-network associated with APE1 (Fig. 1); then, we linked this analysis to additional cancer/biological databases with the aim to provide a more complete picture of the APE1 biological roles in both cancer and cellular biology.

### APE1-PPI network construction and analysis

**Figure 3:**
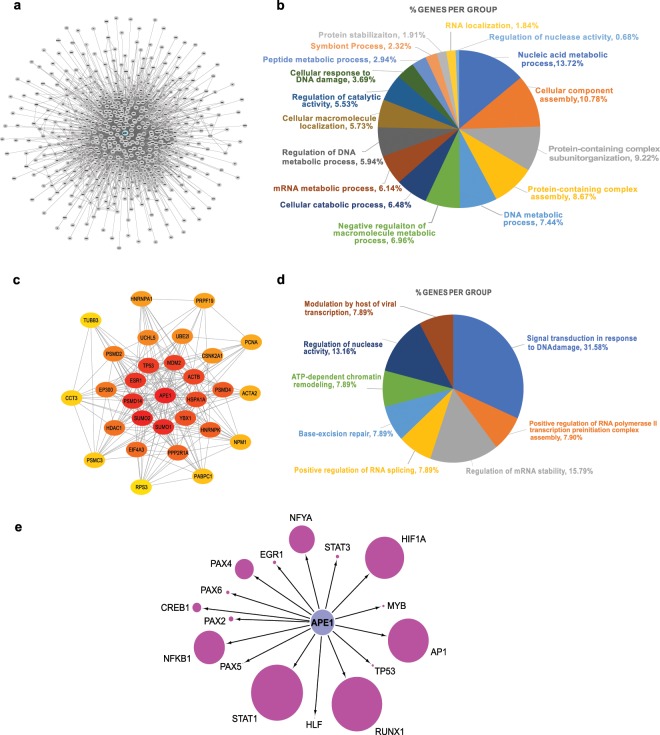
Bioinformatics characterization of the APE1 interactome. (**A**) Global APE1 Protein-Protein Interaction Network. (**B**) Functional annotation of the global network based on Gene Ontology - Biological Process terms (p < 0.05). In the pie chart, the percentage of the proteins/genes enriched in the group of pathways is shown. (**C**) Top 30 hubs of the APE1-PPI network, based on global metric, betweenness centrality. Color shades represent the significance of the hub, with red color as the most significant and yellow color as the least. (**D**) Functional annotation of the Top 30 hubs based on Gene Ontology - Biological Process terms (p < 0.05). In the pie chart, the percentage of the genes enriched in the group of pathways is shown. (**E**) Transcriptional regulatory network of the APE1 interactome. Node size represents the number of putative binding sites identified by the LASAGNA-Search 2.0 tool in the promoters (−2500, -1nt from the TSS) of the APE1 interactome genes for 16 transcription factors that are modified by APE1 redox activity or use APE1 as a co-factor.

The APE1-interacting partners from this and other investigations (n = 535) were used to establish the APE1-PPI network. Direct and/or indirect interactions between these molecules were retrieved by the InWeb_InBioMap web tool, which is a large data compendium for high-quality PPI networks. Afterwards, the undirected PPI network, representing the interactome of APE1, was constructed with 511 nodes (24 proteins were not recognized by the tool) and 3934 edges (Fig. 3A). The resulting network was visualized and analyzed by using the Cytoscape software and its packages^38^. The initial analysis of the network was carried out by performing functional enrichment analysis for terms belonging to the “Gene Ontology - Biological Process” database, using the ClueGO tool with standard parameters to identify enriched pathways on the basis of the network’s gene frequency in each pathway (n = 383, 75%). Based on this analysis, 109 genes were enriched in the group of pathways called “DNA metabolic process” (7.4% genes per group), 90 genes were enriched in the group of pathways called “mRNA metabolic process” (6.1% genes per group), 54 genes were enriched in the group of pathways called “DNA damage response” (3.7% genes per group) and 27 genes were enriched in the group of pathways called “RNA localization” (1.8% genes per group) (Fig. 3B and Supplementary Table S4). These results clearly confirmed the involvement of APE1 and its interacting partners in processes involved in RNA (with particular emphasis on mRNA), DNA and protein metabolism/stability, supporting our previous findings^4,12^.

The complex PPI-network containing 511 nodes was then studied in order to focus on its most important elements; this was done through a hub analysis based on global metric, betweenness centrality. In graph theory, betweenness centrality is a measure of centrality in a graph based on the shortest paths; therefore, it represents the degree to which nodes stand between each other. As a result, the top 30 hub nodes were identified and extracted from the main APE1-PPI network as hub-subnetworks (Fig. 3C). This hub module was then analyzed with ClueGO to understand the specific roles of these genes in biological processes (Fig. 3D and Supplementary Table S4). The predominant enrichment was observed for: i) 12 hub genes involved in the group of pathways named “signal transduction in response to DNA damage” (31.6% genes per group); ii) 6 hub genes enriched in the group of pathways called “regulation of mRNA stability” (15.8% genes per group); iii) 3 central hub genes (HSPA1A, PRPF19 and PSMD4) involved in the group of pathways related to “positive regulation of RNA splicing”, confirming previous interactomic results^4^. Globally, these results indicate the significant involvement of the APE1-PPI network in RNA and DNA metabolism, showing that APE1 acts as the central hub connecting different subnetworks with diverse functions.

### Role of APE1 in the transcriptional regulation of the APE1-PPI network

Among its multifunctional biological roles, APE1 is also known as transcriptional and post-transcriptional regulator. On the one hand, APE1 can exert its nuclear redox control function on several transcription factors (TFs), modulating their activity and, hence, gene expression^39^. On the other hand, it can also act at the RNA level through its ability in binding to transcripts, thus affecting corresponding stability and processing^4,10^. Therefore, we can hypothesize that APE1 may contribute to gene expression regulation of elements of its own PPI. In order to check whether APE1 could be involved in the former activity, we performed a TFBS motif discovery analysis with the LASAGNA-Search 2.0 tool^40^ on the promoters of the above-mentioned 531 genes representing the APE1 interactome. This allowed to verify and quantify the presence of enriched putative binding sites for more than a hundred TFs (Supplementary Table S5). In particular, we identified putative sites for 16 TFs that are known to be stimulated by the APE1 redox activity or to regulate gene expression by using APE1 as a co-factor (*e.g*. AP1, NF-κB1, HIF1α and members of the STAT family) (Fig. 3E). Some of these TF binding sites (*e.g*. PAX5 sites) were not highly abundant in the obtained results but, since they are known to regulate gene expression in a tissue-specific manner, we believe that the HeLa cell model used in the present work could not represent the most suitable one to study their effect. We also compared the list of APE1-PPI partners identified in the present work with that of the RNA molecules bound by APE1 (n = 1015), which we previously had defined through RIP-seq experiments^4,12^; this analysis demonstrated that 42 genes, that interact with APE1 at the transcript level, are also part of the APE1-PPI (p-value = 0.01, Supplementary Fig. S3). We finally integrated these data with the results of a microarray differential gene expression profiling analysis performed in siAPE1 HeLa cells^12^. Interestingly, we found that 55 genes, belonging to the APE1-PPI network, were differentially expressed (absolute log fold change ≥1, adj. p-value ≤ 0.05), as well as 45 other transcripts originating from the RIP-seq experiment, globally accounting for 95 unique genes (p-value = 0.0001, Supplementary Fig. S3). Altogether, these results strongly support the possibility that APE1 may contribute to the regulation of the expression of the majority of its own PPI genes.

### Correlation between APE1 and its protein-binding partners at the gene expression level

Correlated expression of differently associated genes is often observed being a common feature and an important booster of the transformation process^41^, even though a perfect correlation of gene expression at mRNA and protein levels is not always observed^42^. However, data described in the previous section provided interesting evidences suggesting that the integration of proteomic and transcriptomic data, correlated by common biological functions, may highlight the potential involvement of subsets of genes in neoplastic transformation.

**Figure 4:**
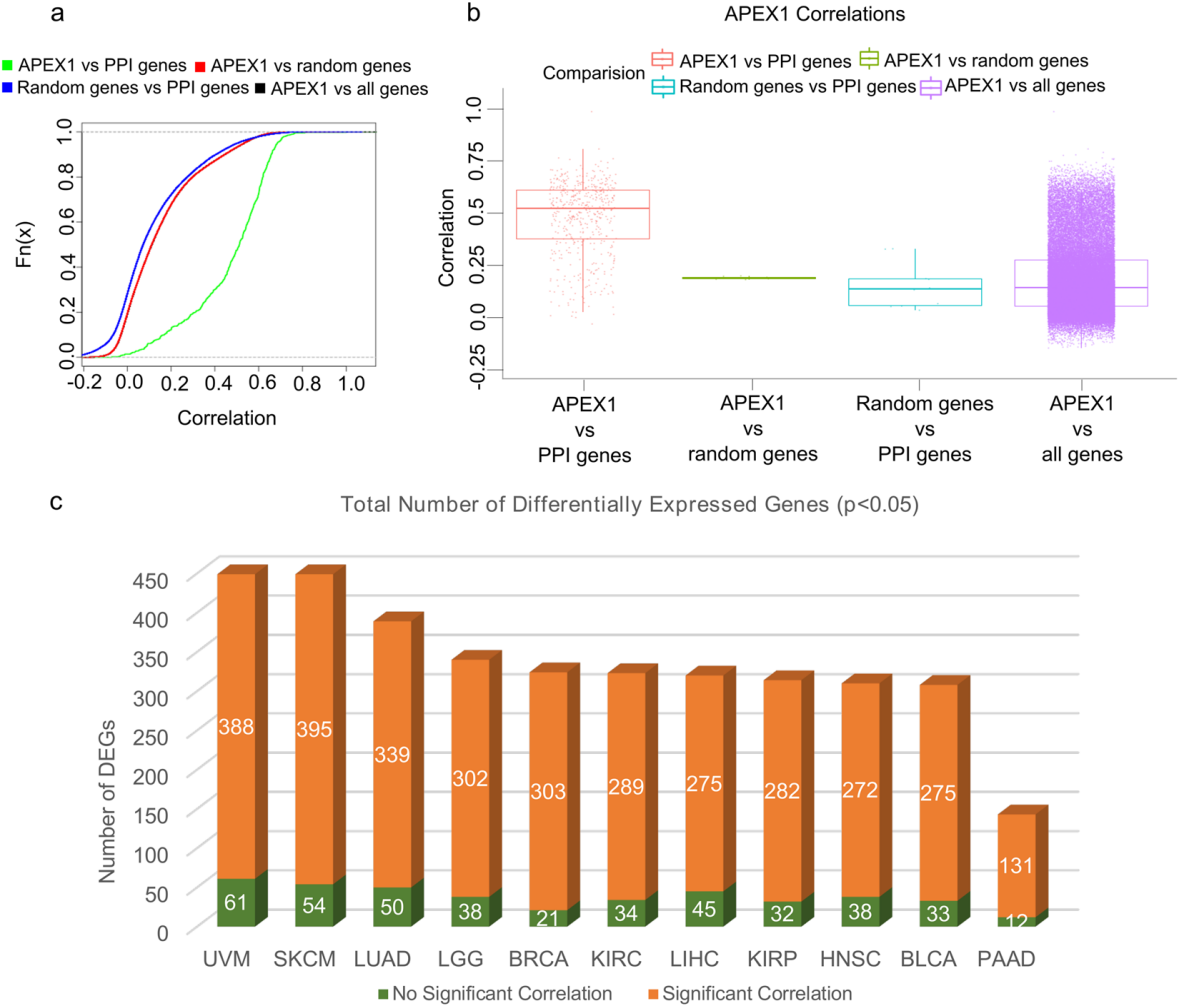
Differential gene expression and correlation analysis of APE1-interacting partners in the TCGA datasets. (**A**) Empirical cumulative distribution function (ECDF). Empirical cumulative distribution curves of the correlation of the gene expression profiles of APEX1 and APEX1-PPI network genes in the TCGA datasets or in the control groups. Green line: APEX1 expression versus the PPI network genes; red line: APEX1 expression versus 100 sets with the same size of the PPI gene set composed by random genes; blue line: 100 random genes expression versus the PPI network genes; black line: APEX1 expression versus all genes. Note that the black line is nearly superimposed to the red line and, for this reason, almost hidden by it. (**B**) Box-plots of the same data. The average correlations for all the control groups are statistically significantly different (p < 0.005) with respect to the APEX1-PPI correlation. (**C**) Total number of differentially expressed genes (*p* < 0.05, absolute log fold change difference > 1) across the top 11 TCGA datasets having the highest number of up- and down-regulated genes. In the bar chart, the number of genes having significant correlation (absolute (PCC) >0.6, *p* < 0.05) with the expression of APE1 is shown in orange, while the others are shown in green.

**Figure 5:**
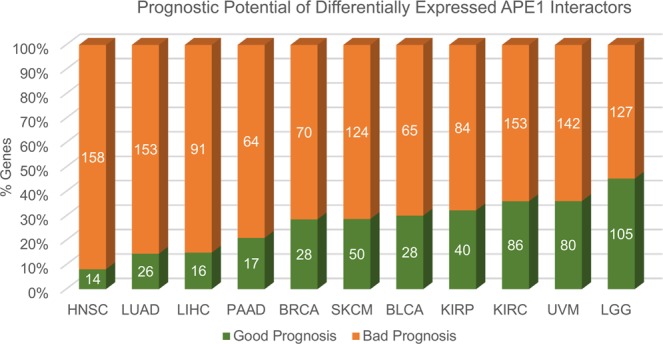
Survival analysis of APE1-interacting partners in the TCGA datasets. For each cancer type, bar plots represent the percentages and the total number of genes having significant (*p* < 0.05) bad or good prognosis, shown in orange and green color, respectively.

In order to understand if a relationship between APE1 and its protein interaction partners may be observed also at the gene expression level, we analyzed the corresponding correlations in the Genomic Data Commons (GDC) RNA-Seq tumor database. In more than 11,000 tumor sample datasets, we observed that the gene expression correlation between APE1 and its PPI-network elements is higher (p-value < 10^15^) than that with respect to: (i) all the genes, (ii) random-gene datasets (PPI size) or iii) random genes *vs* the APE1-PPI network (Fig. 4A,B). By using the same dataset, gene expression data for the genes coding the 531 interactors of APE1 were obtained for 33 different tumor types described in TCGA (Supplementary Table S6) as well as for the associated normal tissues. Further analyses were performed across the top 11 TCGA cancer datasets having the highest number of bad prognostic genes (see below) that were up- and down-regulated (*p* < 0.05, absolute log fold change >1) (Figs. 4C, 5 and Supplementary Table S6). According to the results of the differential gene expression analysis, more than half of the APE1 interactors were found to be differentially expressed in many datasets (Fig. 4). The gene expression of APE1 across these datasets was used to calculate the Pearson correlation existing between APE1 and its interactors. A very high percentage of differentially expressed genes (DEGs) in those datasets was found to have significant (*p* < 0.05) correlation with APE1 gene expression (Fig. 4C). Altogether, these results demonstrated the existence of a very strong orchestration in the expression of APE1 and of its interacting proteins in the aforementioned cancer types, suggesting the existence of common pathways of transcriptional regulation for APE1-PPIs in cancer development.

### Upstream regulators analysis

**Figure 6:**
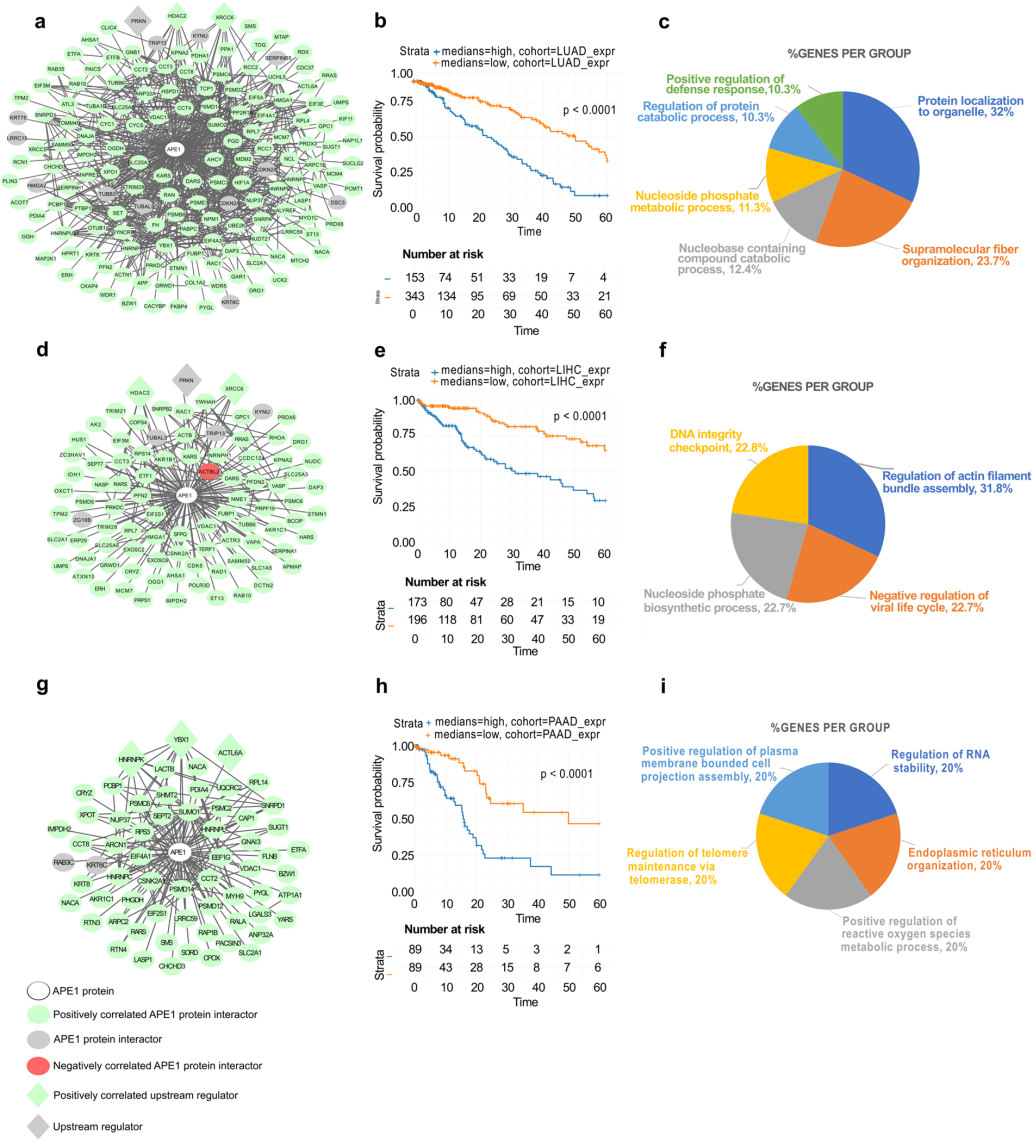
The most relevant cancer-specific prognostic subnetworks in the APE1 interactome. (**A,D,G**) Bad Prognosis networks of the LUAD (**A**), LIHC (**D**) and PAAD (**G**) datasets formed by the protein interactors of APE1. Circular nodes represent the interactors of APE1, while diamond nodes represent the top 3 upstream regulators of the network. APE1 is symbolized with the white color as the central node. Significant negatively correlated nodes are symbolized with the red color (PCC < −0.6), while the nodes having significant positive correlation (PCC >0.6) are symbolized with the green color. Upstream regulators are symbolized with the purple color and the rest of the nodes are symbolized with the grey color. (**B**,**E**,**H**) Survival probability of the patients having high and low median expression of the gene signatures forming the overall networks, as represented by Kaplan-Meier plots. (**C**,**F**,**I**) Functional annotation of the Bad Prognosis networks based on Gene Ontology - Biological Process terms (*p* < 0.05). In the pie chart, the percentage of the genes enriched in the pathways is given next to the enriched terms.

With the aim of ascertaining the role of these gene modules in more detail, an upstream regulators analysis was performed using the TRANSPATH tool within the geneXplain 4.11 web platform. For each TCGA cancer dataset, the list of significant DEGs was analyzed, and the three molecules having the lowest Ranks sum were retained (Table 1) as the ones having the best probability of being the master regulators of that network. These results strengthened, on the one hand, the general role of the modules in RNA and DNA repair mechanisms. In fact, XRCC6 (master regulator of 5 datasets) and DDB1 (master regulator of 3 datasets) are proteins associated with the DNA repair process^43,44^. YBX1, another highly represented master regulator (KIRC, KIRP and PAAD), is known to act as both an RNA- and a DNA-binding protein, as well as being involved in miRNA processing^45^. However, what really equated all these regulators was their role in the apoptotic, proliferative and resistance pathways, as shown in Table 1. We also evaluated the correlation of the expression profiles of APEX1 and of these regulators in each bad prognostic network (Fig. 6 and Supplementary Figs. S4–S11, data is represented according to a color code).

**Table 1:** APE1-PPI bad prognostic signatures top regulators analysis.

Dataset	Top1	Top1 Bibliography	Gene Symbol Description	Top2	Top2 Bibliography	Gene Symbol Description	Top3	Top3 Bibliography	Gene Symbol Description
HNSC	XRCC6	(1) (2) (3)	X-ray repair cross-complementing protein 6	PRKDC	(1) (2) (3)	Protein kinase, DNA-activated, catalytic subunit	TERF2	(1) (2) (3)	Telomeric repeat binding factor 2
KIRC	PRKCD	(1) (2) (3)	Protein kinase C delta type	PPP1CB	(2)	Protein phosphatase 1 catalytic subunit beta	YBX1	(1) (2) (3)	Y-Box binding protein 1
LUAD	PRKN	(2)	Parkin RBR E3 ubiquitin protein ligase	HDAC2	(1) (2) (3)	Histone deacetylase 2	XRCC6	(1) (2) (3)	X-ray repair cross-complementing protein 6
UVM	SMYD2	(1) (2) (3)	SET and MYND domain containing 2	KDM1A	(1) (2) (3)	Lysine demethylase 1 A	XRCC5	(1) (2)	X-ray repair cross-complementing protein 5
LGG	SETD7	(1) (2) (3)	SET domain containing 7, histone lysine Methyltransferase	KDM1A	(1) (2) (3)	Lysine demethylase 1A	CUL1	(1) (2)	Cullin-1
SKCM	DDB1	(1) (2) (3)	Damage specific DNA binding protein 1	PTP4A3	(1) (2) (3)	Protein tyrosine phosphatase 4A3	XRCC6	(1) (2) (3)	X-ray repair cross-complementing protein 6
LIHC	PRKN	(2)	Parkin RBR E3 ubiquitin protein ligase	HDAC2	(1) (2) (3)	Histone deacetylase 2	XRCC6	(1) (2) (3)	X-ray repair cross-complementing protein 6
KIRP	YBX1	(1) (2) (3)	Y-Box binding protein 1	PTP4A3	(1) (2) (3)	Protein tyrosine phosphatase 4A3	TCF21	(1) (2) (3)	Transcription factor 21
BRCA	DDB1	(1) (2) (3)	Damage specific DNA binding protein 1	XRCC6	(1) (2) (3)	X-ray repair cross-complementing protein 6	IDE	(1) (2)	Insulin-degrading enzyme
BLCA	DDB1	(1) (2) (3)	Damage specific DNA binding protein 1	XRCC5	(1) (2)	X-ray repair cross-complementing protein 5	TRIM28	(1) (2) (3)	Tripartite motif containing 28
PAAD	YBX1	(1) (2) (3)	Y-Box binding protein 1	ACTL6A	(1) (2)	Actin-like protein 6A	HNRNPK	(1) (2) (3)	Heterogeneous nuclear ribonucleoprotein K

GeneXplain identification of the Top 3 putative master regulators (ranked by ascending Ranks sum) of bad prognostic genes in the 11 selected TCGA cancer datasets. Numbers in brackets refer to the presence of bibliographic evidence associating upstream regulators with proliferation (1), apoptosis (2) and resistance (3), respectively. For TCGA abbreviations, see Supplementary Table S6; for all the references Supplementary Table S7.

Afterwards, upstream regulators analysis was performed also on the global APE1-PPI network to widen our understanding of its functions. The resulting top 10 master regulators are provided in Supplementary Table S8. Interestingly, we observed that some of these master regulators (i.e. NUAK1, KDM1A, UBE2D1, RBX1 and UBE2M) were known to be associated with the p53 signaling pathway^46–51^.

### Definition of a bad-prognostic APE1 signature in cancers

With the aim of evaluating the real impact of APE1-interacting partners in TCGA cancer datasets, survival analyses were performed using the RTCGA Bioconductor package. The genes that were differentially expressed in a statistically significant manner (*p* < 0.05, absolute log fold change >1) between tumor and normal tissues were analyzed; Kaplan-Meier plots were obtained for each gene in each dataset, allowing to define good and bad prognosis gene signatures on a per cancer basis (Supplementary Table S4). The distribution of the genes with respect to significant good or bad prognosis (*p* < 0.05) in those cancer datasets is represented in Fig. 5.

In order to focus our attention on the most relevant cancer types, we based our bioinformatic analysis on the eleven datasets having the highest number of bad prognostic genes. Among those, we were interested in particular in liver (LIHC)^52–56^, lung (LUAD)^57–60^ and pancreatic (PAAD)^61–65^ cancer datasets, as the essential role of APE1 in the tumorigenic processes of these cancer types is already well established. Notably, the LUAD dataset was the third having the highest number of bad prognostic genes (n = 153). We then represented these genes as a subnetwork of the APE1-PPI network having correlation information with the following color code: green for significant positive correlation (PCC >0.6), red for significant negative correlation (PCC <−0.6) and grey for no correlation (Fig. 6A). In the network, the top 3 master regulators were also represented with diamond-shaped nodes. Afterwards, we specifically studied the survival outcomes of patients with high or low expression of the bad prognosis signature, using the median log fold change value of the genes in the network. The Kaplan-Meier plot (Fig. 6B) clearly shows that high expression of the genes in the bad prognostic network was associated with significant (*p* < 0.0001) lower survival probability. The biological processes dominantly enriched in this network were as follows: nucleic acid metabolism, protein transporters and arrangements to form complex subunits that have polymerized to generate fiber-shaped structures (Fig. 6C and Supplementary Table S4).

Because of the strong interest in APE1 as a prognostic factor in liver^52–56^ and pancreatic cancers^61–63^, we applied the same representation also to the LIHC (Fig. 6D–F) and PAAD datasets (Fig. 6G–I) with a total number of 91 and 64 bad prognostic genes, respectively. The bad prognostic signatures in these networks highlighted again a significant involvement of DNA- and RNA-related pathways (Figs. 6F–I; Supplementary Table S4). In general, the results of survival analysis emphasized the importance of the APE1-PPI network for real clinical data. Specifically, 84.3% of the genes (n = 451) present in the APE1-PPI network was associated with bad prognosis in one or more cancer datasets.

### APE1-inhibitors sensitize cancer cells to mitochondrial toxicant Rotenone

**Figure 7:**
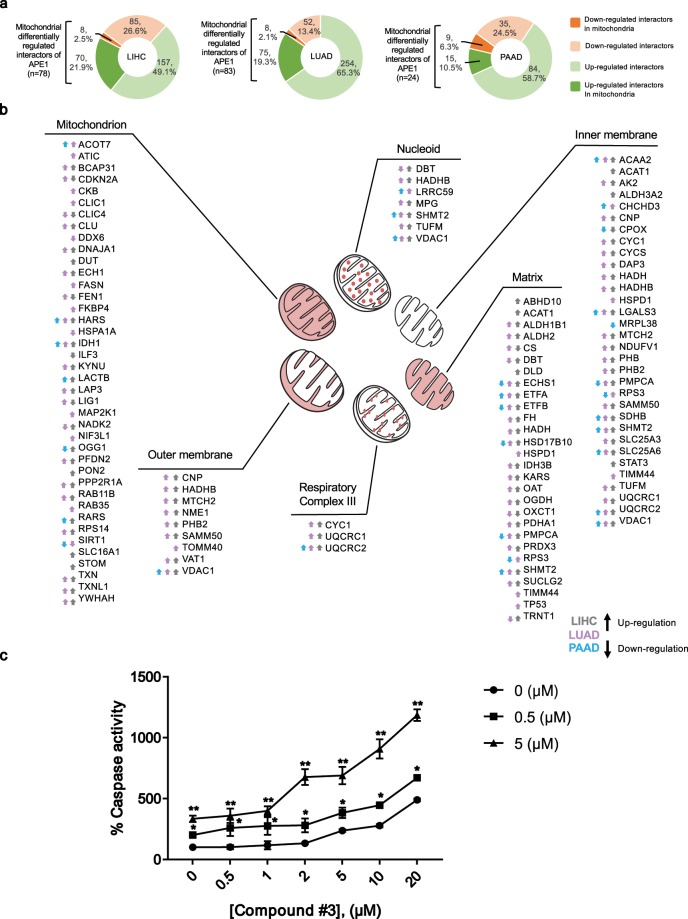
Differentially expressed APE1-PPIs in LIHC, LUAD and PAAD datasets point to the relevance of mitochondrial pathways impairment in cancer. (**A**) Differentially expressed APE1 interactors with mitochondrial localization. The colour codes are as follows: bright green colour for up-regulated DEGs that can localize to mitochondria, bright orange colour for down-regulated DEGs that can localize to mitochondria, light green and light orange colours for up- and down- regulated DEGs that do not localize to mitochondria, respectively. (**B**) Localization of DEGs in the mitochondrial compartments. Specific mitochondrial sub-compartmentalization of DEGs is shown. For each location, the global expression trend and dataset information is given with arrow and colour codes. Upside arrow represents up-regulation, while downside arrow represents down-regulation. The colour of the arrows represents the datasets as follows: grey colour for LIHC, purple colour for LUAD and blue colour for PAAD. (**C**) Inhibition of APE1 endonuclease activity in combination with treatment with rotenone sensitizes HeLa cells to apoptosis. Cells were treated with Compound #3 in the absence or presence of rotenone (0.5 and 5 µM), for 24 h. The Apo-ONE assay was used to quantify relative levels of apoptosis. The activities of caspases 3/7 were examined using a fluorescence-based assay. Data were normalized on untreated cells and represent the means ± SD of three independent experiments. Asterisks represent a significant difference with respect to cells treated with Compound #3 alone. Data were evaluated statistically by two-tails Student t-test.

In order to refine our study and to provide an additional level to the functional characterization of APE1-PPI, we finally performed a comparative bioinformatics analysis to define the cellular localization of DEGs, as obtained from the LIHC, LUAD and PAAD datasets. As shown in Fig. 7A, 24.4% of the DEGs in LIHC, 21.4% of the DEGs in LUAD and 16.8% of the DEGs in PAAD represented APE1-PPIs that can also localize to mitochondria. Interestingly, 89.7%, 90.4% and 62.5% of these DEGs were observed to be upregulated in the corresponding TCGA datasets. The mitochondrial compartments and expression trends associated with these DEGs are shown in Fig. 7B.

With the aim to support APE1 involvement in mitochondrial functionality, we performed a caspase activity assay upon treatment with Compound #3, an APE1 endonuclease inhibitor^66^, in combination with rotenone, a well-known mitochondrial respiratory chain inhibitor^67^. HeLa cells were treated with different concentrations of Compound #3 in the presence/absence of rotenone (at the doses of 0.5 μM and 5 μM) for 24 h (Fig. 7C) (Supplementary Fig. S12). HeLa cells treated with the combination of both compounds resulted more sensitive to apoptosis, demonstrating the existence of a synthetic lethality relation between APE1 and mitochondrial activity, which may be further explored for designing novel anticancer strategies.

## Discussion

The multifunctional DNA repair protein APE1 is a central enzyme in the BER pathway, and it has also been involved in the regulation of cellular response to genotoxic damage via direct and indirect mechanisms. In addition to the primary roles of APE1 in DNA damage repair, emerging evidences indicate that APE1 may also control RNA metabolism processes and thus gene expression^4,13,68^. In order to better clarify these novel functional aspects, we used well-established systems biology methods as optimal procedures to dissect/identify newly arising roles and possible mechanisms involving APE1. In this study, we focused on interactomic information obtained from previous studies^4,12^ as well as from novel experiments, which were performed using sensitive mass spectrometry technologies applied to the analysis of subcellular compartments. This allowed defining a final list containing 535 APE1-PPI elements that were finally subjected to a deep bioinformatics investigation.

Starting from this comprehensive list of proteins, the identification of direct and/or indirect interactions among APE1-binding partners was deduced, and a direct/indirect PPI network representing the global interactome of APE1 was constructed. This network, which was composed of 511 nodes and 3934 edges, retained a level of complexity that hampered the full understanding of the specific properties of each node. Therefore, a hub analysis was accomplished to define the top 30 nodes that were crucial for the communication of the whole network. Specifically, hub analysis was performed on betweenness centrality, a global metric that determines the involvement of each node in the information flow within the network. As a consequence, this method allowed the identification of the most informative proteins (hubs) for optimized therapeutic targeting in specific tumors (based on further bioinformatic analyses reported below). Among the most important hubs, APE1, SUMO1, SUMO2, TP53, ESR1, MDM2, PSMD4 and ACTB are worth mentioning (Fig. 3C). The resulting hub module was then analyzed with the Cytoscape plugin ClueGO to understand the role of these proteins in biological processes; the obtained results pointed to the involvement of these proteins mainly in DNA damage, mRNA stability and RNA splicing (Fig. 3D and Supplementary Table S4), indicating how these pathways were intertwined through the central role of APE1.

These data corroborated an emerging evidence in tumor biology; in fact, many DNA repair proteins are associated with those involved in RNA metabolism, thus proving a substantial role of the corresponding interactome networks in determining their non-canonical functions, which impact on gene expression in tumor cells^3^. These considerations emphasized the importance of understanding the regulatory behavior of APE1-interacting partners in various cancer datasets. To achieve this, the differential expression profiles and the gene expression correlations of APE1 and its interacting partners were calculated through the analysis of 33 TCGA datasets. The top 11 datasets with the highest number of differentially expressed genes, together with their correlation information, are represented as a bar chart in Fig. 4C. A very high percentage of differentially expressed genes was observed to have a significant prognostic potential, which was indicative for the relevance of these gene sets for related cancers (Fig. 5). These 11 gene sets were then subjected to survival analysis for a better understanding of their role according to a clinical perspective. LIHC, LUAD and PAAD networks, which were composed of bad prognostic genes in high percentages, confirmed previous studies on the important role of APE1 in these cancers^52–55,57–65^. Kaplan-Meier estimation was used to calculate survival probability associated with these genes; the ones having significantly lower survival prognosis (*p* < 0.05) were used to create cancer-specific PPI subnetworks (Fig. 6 and Supplementary Figs. S4–S11). These cancer-specific bad prognostic network signatures unveiled potential protein interactor targets to be further probed by novel therapeutic approaches.

In addition, the top 11 datasets were used to perform the upstream regulators analysis for wider understanding of the regulatory events acting on the gene sets and the top 3 master regulator genes, as well as their involvement in the apoptotic, proliferative and resistance pathways, were characterized. Out of 11 datasets, XRCC6 was found to be one of the top 3 master regulators in 5 datasets (*i.e*. HNSC, LUAD, LIHC, SKCM and BRCA), together with its interacting partner XRCC5, which was the master regulator of 2 datasets (UVM and BLCA). XRCC6 and XRCC5 are coding the proteins Ku70 and Ku80, which form a molecular heterodimer involved in the initial step of the non-homologous end joining (NHEJ) pathway. Ring-shaped Ku complex directs DNA-dependent protein kinase catalytic subunit (DNA PKcs coded by PRKDC/XRCC7) to the DNA ends, and triggers its kinase activity for DNA repair^69^. Interestingly, PRKDC was found among APE1 interactors as a component having a high correlation and bad survival prognosis in various datasets. It was previously reported that genetic variants of PRKDC play an important role in splicing regulation, causing mRNA instability^70^. PRKDC and XRCC6 play an important role in the suppression of chromosomal rearrangements and in the maintenance of genome integrity, along with a significant function in the recognition and repair of double strand breaks^70^. Therefore, their roles were studied in various cancers, such as breast^71,72^, glioma^73^, renal^74^, hepatocellular^75,76^, digestive^77^, bladder^78^ and lung^70^ cancer. On the other hand, Ku complex is known to interact with RECQL4 and to form a macromolecular assembly promoting NHEJ. A well-known RECQL4-binding partner, namely DDB1^79^, was found to be one of the top 3 master regulators of 3 datasets (SKCM, BLCA and BRCA). DDB1 has been reported to be involved in the damage recognition step of the BER pathway^80^, and to be correlated with a high risk when down-regulated in head and neck squamous cell carcinoma^81^. The oncogenic transcriptional factor of RECQL4, namely YB1 (coded by YBX1), was here recognized as an interacting partner of APE1 and a master regulator observed in KIRC, KIRP and PAAD datasets. YB1 was found to be overexpressed in various cancer types and frequently associated with poor outcome and chemotherapy resistance^82,83^. It has also been reported to act as both an RNA- and DNA-binding protein, and as a component involved in miRNA processing^45,84^. These findings underline the important involvement of APE1-centered prognostic networks mainly in DNA repair, with the association of RNA metabolism in various cancer types.

Among the remaining top regulators identified in this study, we also found some important genes that might give some clues about the general involvement of the APE1 interactome in the p53 signaling pathway. In particular, some of the top 10 master regulators that were associated with the p53 signaling pathway (*i.e*. NUAK1, KDM1A, UBE2D1, RBX1 and UBE2M) deserved particular attention. For example, NUAK1 is known to be involved in DNA damage response by phosphorylating p53 and participating in the transcriptional regulation of the *CDKN1A* promoter^46^. Since NUAK1 is phosphorylated by AKT^47^, it has been hypothesized that this protein could be involved in acting between ATR and CDKN1A in response to low doses of UV irradiation^48^. Analogously, the master regulator KDM1A (LSD1) was hypothesized to be a potential therapeutic target for the estrogen-regulated type I endometrial cancer because of its crucial role in the LSD1/cyclin D1/PI3K/AKT feedback loop^49^. On the other hand, Zhou and colleagues recently reported that UBE2D1 facilitated the growth of hepatocellular carcinoma *in vitro* and *in vivo* by decreasing the p53 protein level in an ubiquitin-dependent manner^50^. Interestingly, the master regulators UBE2M and RBX1 were also reported to have up-regulation together with other neddylation enzymes to highlight overexpression of the neddylation pathway in HCC^51^. In conclusion, our analysis of the global network indicated the relation of APE1 and its interactors with DNA repair mechanisms, and the possible involvement of the p53 signaling pathway, as confirmed by the identification of p53 binding sites among the significantly enriched TFBS located in the promoters of APE1-PPI genes. However, what associated the highest number of top regulators was their role in the apoptotic, proliferative and resistance pathways.

In order to further characterize the LIHC, LUAD and PAAD TCGA datasets, the corresponding DEGs were examined also considering their subcellular locations. As a result, 24.4% of DEGs in LIHC, 21.4% of DEGs in LUAD and 16.8% of DEGs in PAAD datasets pointed to the involvement of APE1-PPIs in mitochondria functionality (Fig. 7). For example, the bad prognostic gene VDAC1 (voltage dependent anion channel 1), which is a multifunctional mitochondrial protein and an important regulator of cancer cell fate through its metabolic and energetic functions^85,86^, was commonly up-regulated in all 3 datasets. Similarly, many other mitochondria-resident APE1-PPIs were found to be commonly up-regulated in the analyzed datasets. Among these, it is worth mentioning: (i) SHMT2 (serine hydroxymethyltransferase), that mainly localizes to the matrix, nucleoid and inner membranes, and is known to be targeted by c-myc for cell survival, with various studies confirming its bad prognostic power in different cancer types^87–91^; (ii) pro-apoptotic protein SLC25A6 (mitochondrial ADP/ATP carrier-3, AAC3)^92,93^; (iii) ROS regulating respiratory complex III protein UQCRC2 (ubiquinol-cytochrome c reductase complex core protein 2)^94^; (iv) respiratory complex II protein SDHB (succinate dehydrogenase B)^95^; v) fatty acid β- oxidation proteins ETFA (electron transfer flavoprotein subunit alpha)^96,97^ and ACAA2 (acetyl-CoA acyltransferase 2)^98^; vi) bad prognostic multifunctional LGALS3 (galectin-3) protein^99^; (vii) autoimmunity protein HARS (histidyl-tRNA synthetase)^100^; (viii) oxidative damage control protein IDH1 (isocitrate dehydrogenase 1)^101^. Additional matrix proteins were found being up-regulated in LIHC and LUAD, while down-regulated in PAAD; they included fatty acid β-oxidation proteins ECHS1 (enoyl coenzyme A hydratase short chain 1)^102^ and ETFB (electron transfer flavoprotein subunit beta)^103^, multifunctional protein 17β-HSD10 (17β-hydroxysteroid dehydrogenase type 10, encoded by HSD17B10)^104^ and mitochondrial protein processor PMPCA (mitochondrial-processing peptidase subunit alpha)^105^. The relevance of APE1 function within mitochondria in tumor cells has recently been highlighted by our^106^ and other publications^107^, emphasizing a pivotal role for APE1 in mitochondrial-mediated signalling in cancer cells, thus opening new perspectives in cancer therapy.

Testing on a limited set of APE1-interacting partners, we previously observed that the APE1-interactome is dynamically regulated during genotoxic stress conditions^4^ with an enrichment of proteins involved in BER, while losing interaction with typical proteins involved in RNA metabolism. It would be interesting to extend this study to the whole APE1-PPI network described here. On the same track, it would be interesting to specifically evaluate the contribution of DNA and RNA molecules in modulating the APE1-PPI network dynamics. Information on the latter aspects may be obtained by characterizing the APE1-interactome before and upon the enzymatic removal of RNA and DNA. In conclusion and for the sake of clarity, we must state that this work is hypothesis generating and future studies will be needed to assess the function of APE1 in the protein complexes we discovered. Our current work is actually focused along these lines.

## Materials and Methods

### Cell line and materials

Inducible HeLa cell clones silenced for endogenous APE1 and reconstituted with the ectopic FLAG-tagged APE1 form were used^11^. HeLa cell clones were grown in Dulbecco’s modified Eagle’s medium (Invitrogen, Monza, Italy) supplemented with 10% v/v fetal bovine serum (Euroclone, Milan, Italy), 100 U/ml penicillin, 10 μg/ml streptomycin sulphate, 3 μg/ml blasticidin, 100 μg/ml zeocine, 400 μg/ml geneticin (Invitrogen), and cultured in a humidified incubator containing a 5% CO_2_ atmosphere, at 37 °C. For inducible APE1-shRNA experiments, doxycycline (1 μg/ml) (Sigma-Aldrich, St. Louis, MO) was added to the cell culture medium and cells were grown for 10 days. JHH-6 cells (undifferentiated hepatocellular carcinoma)^31^ were cultured in William’s medium E (Sigma-Aldrich), while A549 (adenocarcinomic human alveolar basal epithelial cells)^32^ cells were cultured in RPM1 (Euroclone); both cell cultures were supplemented with 10% v/v fetal bovine serum, 100 U/ml penicillin, 10 μg/ml streptomycin sulphate. All cell lines were tested for mycoplasma contamination (N-GARDE Mycoplamsa PCR Reagent, Euroclone).

### Preparation of cell extracts and co-immunoprecipitation

Immunoprecipitation studies were carried out with whole cell extracts, and nuclear or cytoplasmatic subfractions of HeLa cell clones as already reported^11,108^ (see Supplementary Material and Methods for details).

### Immunofluorescence confocal and Proximity Ligation analyses

Immunofluorescence procedures and Proximity Ligation Assay (PLA) (Duolink, Sigma-Aldrich) were carried out as described earlier^4^. PLA was performed following the manufacturer’s instructions. Cells were visualized through a Leica TCS SP8 confocal system (Leica Microsystems GmbH, Germany). See Supplementary Material and Methods for the list of the antibodies used.

### Antibodies used and Western blotting analysis

For Western blotting analyses, cell lysates were resolved on 12% T SDS-PAGE, transferred onto nitrocellulose membranes (Amersham^TM^ Protran^TM^, GE Healthcare) and probed with the indicated antibodies (see Supplementary Material). The corresponding secondary antibodies labeled with IR-Dye (anti-rabbit IgG IRDye 680 and anti-mouse IgG IRDye 800) were used. Detection was performed with the Odyssey CLx Infrared imaging system (LI-COR GmbH, Germany). Protein bands were quantified using Odyssey software (Image Studio 5.0). Original uncropped images of western blots used in this study can be found in Supplementary Fig. S13.

### Proteomic analysis

Immunopurified proteins from whole, nuclear and cytoplasmic cell extracts of HeLa cell clones expressing ectopic APE1 FLAG-tagged protein or stably transfected with the empty vector^12^ (SCR) were analyzed in parallel by 12% T SDS-PAGE. As additional control experiment, identical cell extracts from HeLa cells expressing APE1 FLAG-tagged were also co-immunoprecipitated with a resin lacking the FLAG antibody (res). After staining with colloidal Coomassie blue, whole gel lanes from all samples were cut into 12 slices, minced and washed with water. Corresponding proteins were separately *in-gel* reduced, *S*-alkylated with iodoacetamide and digested with trypsin, as previously reported^109^ and subjected to mass spectrometry analysis as detailed in Supplementary Material and Methods. A careful filtration for false positives ascertained in control samples (SCR and res) from whole, nuclear or cytoplasmic cell extracts allowed identifying APE1-binding proteins in APE1-FLAG co-immunoprecipitates from the corresponding cell extracts.

### APE1-PPI network construction

The gene list of APE1-interacting partners was used to construct the corresponding PPI network by defining the interactions between the partners using the InWeb_InBioMap tool, applying the suggested parameters^110^. The APE1-PPI network was represented as an undirected graph (*i.e*., nodes and edges symbolize proteins and interactions between them, respectively), and it was visualized via Cytoscape (v3.6.1)^38^. The network enrichment analysis was performed using the ClueGO tool, using standard parameters^111^. The hubs of the network were obtained by using the Cytohubba tool based on the global metric, betweenness centrality^112^.

### Transcription factor binding sites discovery

The FASTA-formatted sequences corresponding to the promoters (−2500,−1 nt from the TSS) of the 531 genes of the APE1 interactome were recovered using the “getfasta” command of the BEDTools toolset^113^. Sequences were analyzed using the LASAGNA-Search 2.0 tool to identify the presence of enriched transcription factor binding sites (TFBS); the matrix-derived model that used JASPAR CORE matrices was selected and the positions of the 145 TF models available were mapped for every promoter (Cutoff p-value: 0.001). For each promoter, the top10 results were then identified and used to provide the global counts of each TFBS in the analyzed dataset. Finally, the putative binding sites for TFs that underwent APE1 redox activity or that played their regulatory activity using APE1 as co-factor were represented as a network using Cytoscape, with node size corresponding to the number of identified binding sites.

### Tumor datasets and differential gene expression analysis

The differential gene expression results from TCGA and normal datasets (GTEX data) for the genes encoding the proteins present in the APE1-PPI network were obtained via the GDC data portal hub (https://portal.gdc.cancer.gov/, last accessed July 2018). The RUVSeq package inside the R/Bioconductor environment was used to eliminate the batch effect coming from the combination of two data sources^114^. In order to better estimate the differentially expressed genes between the tumor and the normal corresponding datasets, we obtained “in-silico empirical” negative controls, *i.e*., the least significantly DE genes based on a first-pass DE analysis performed prior to RUVg normalization^114^. Empirical Distribution analysis, Pearson correlations and Kolmogorov-Smirnov analyses were performed between the gene expression profiles of APE1 and APE1-PPI elements in cancer patients or in the control groups using the *stats* package inside the R/Bioconductor environment. In particular, we compared the correlations of APE1 expression *vs* the PPI network genes expression with respect to: a) APE1 expression *vs* 100 sets with the same size of the PPI genes set composed by random genes; b) 100 random genes expressions *vs* the PPI network genes; c) APE1 expression *vs* all genes.

The analyses of the differentially expressed genes based on GO-CC (Gene Ontology-Cellular Component) was performed using the DAVID annotation tool^115^.

### Survival analysis

For each TCGA dataset, differentially expressed genes (multiple correction adjustment using the Benjamini-Hochberg method, *p* < 0.05; absolute log fold change difference ≥1) corresponding to an interacting partner of APE1 were used to perform survival analysis. Kaplan-Meier plots were drawn using the RTCGA Bioconductor package^116^, which uses maximally selected rank statistics (maxstat) to determine the optimal cutpoint for continuous variables. Division of the samples was done within the 30–70% percentile range of gene expression by the optimal cutpoint value. The Benjamini-Hochberg method was used for p-value correction of Kaplan-Meier plots.

### Upstream regulators analysis

The set of genes corresponding to APE1 interactors having significant differential expression and significant bad survival prognosis was used to define cancer specific bad prognostic subnetworks of APE1 for the top 11 TCGA datasets. For each subnetwork, putative master regulators were identified by the TRANSPATH database (5.1.1.1)^117^ through the geneXplain platform (geneXplain web edition 4.11)^118^. Identified regulators (max radius: 4; Score cutoff: 0.2; FDR cutoff: 0.05; *Z*-score cutoff: 1.0) were sorted ascendingly based on the Ranks sum, reflecting a combination of sorting by Score and by *Z*-score. Upon sorting by Score from the biggest values to the lowest, a rank was assigned to the molecules (the molecule with the highest Score had rank 1). Upon independent sorting by *Z*-Score from the biggest values to the lowest, a rank was assigned to the molecules (the molecule with the highest *Z*-score had rank 1). Afterwards, for each molecule, the ranks upon sorting by Score and upon sorting by *Z*-Score were summed up, and the Ranks Sum was generated. The lower the Ranks sum, the more interesting the candidate molecule was, with good Score and good *Z*-score values. The same analysis was also repeated on the APE1-PPI network regardless of any differential expression analysis, survival probability and cancer type.

### Caspases activity assay

Caspase 3/7 activity levels were measured in a fluorescence-based assay using the Apo-One^®^ Homogeneous Caspase 3/7 assay (Promega Corp., WI, USA). Assays were performed according to the manufacturer’s recommendations. Four-thousands cells were plated onto black 96-well plates and the day after cells were treated with Compound #3^66^ in the presence/absence of rotenone (R8875, Sigma-Aldrich) (0.5 μM and 5 μM), for 24 h. Fluorescence was measured at 521 nm by using a multi-well plate reader (Enspire 2300 Multilabel Reader, PerkinElmer). The values were standardized to wells containing media alone.

## Supplementary information

Supplementary Table S1.

Supplementary Table S2.

Supplementary Table S3.

Supplementary Table S4.

Supplementary Table S5.

Supplementary Table S6.

Supplementary Table S7.

Supplementary Table S8.

Supplementary info.

## Data Availability

The mass spectrometry proteomics data have been deposited to the ProteomeXchange Consortium via the PRIDE^119^ partner repository with the dataset identifier PXD013368. Reviewer account details: Username: reviewer03955@ebi.ac.uk Password: Rh2r9CaX.
