# Supplementary material for: Architecture of The Human Ape1 Interactome Defines Novel Cancers Signatures
Source: Sci Rep. 2020 Jan 8;10:28. doi: 10.1038/s41598-019-56981-z (PMC6949240; doi:10.1038/s41598-019-56981-z)
Supplement: Supplementary file 3 — Supplementary Table S3. [file 41598_2019_56981_MOESM3_ESM.docx]

**Title of the Paper: ARCHITECTURE OF THE HUMAN APE1 INTERACTOME DEFINES NOVEL CANCERS SIGNATURES**

Authors list: Dilara Ayyildiz^1,#^, Giulia Antoniali^1,#^, Chiara D’Ambrosio^2,#^, Giovanna Mangiapane^1^, Emiliano Dalla^1^,Andrea Scaloni^2^, Gianluca Tell^1,^* and Silvano Piazza^3,^*

^1^Laboratory of Molecular Biology and DNA repair, Department of Medicine, University of Udine, p.le M. Kolbe 4, 33100 Udine, Italy; Phone: +39 0432 494311.

^2^Proteomics and Mass Spectrometry Laboratory, Institute for the Animal Production System in the Mediterranean Environment (ISPAAM), National Research Council (CNR) of Italy, via Argine 1085, 80147 Naples, Italy; Phone +39 081 5966006.

^3^Bioinformatics Core Facility, Centre for Integrative Biology (CIBIO), University of Trento, via Sommarive 18, 38123, Povo (Trento), Italy; Phone: +39 0461 283790.

^#^ These authors equally contributed to the present work.

***Corresponding authors**: Silvano Piazza (silvano.piazza@unitn.it) and Gianluca Tell (gianluca.tell@uniud.it)

**Supplementary Table S3.**

**Confirmation of APE1 Interacting partners in literature.**

The literature evidences of some of the APE1 interacting partners found in our analysis.

| **APE1 Interacting Protein** | **Source** | **Reference** |
| --- | --- | --- |
| ACTN1 | PubMed | Antoniali et al. 2017 |
| AICDA | PubMed | Antoniali et al. 2017 |
| ANP32A | HPRD, BioGRID | Fan, Beresford, Zhang, et al. 2003 |
| ANP32C | HPRD | Fan, Beresford, Zhang, et al. 2003 |
| ANPEP | PubMed | Antoniali et al. 2017 |
| APP | BioGRID | Oláh et al. 2011 |
| ARIH2 | BioGRID | Kristensen, Gsponer, and Foster 2012 |
| ASCL2 | BioGRID | Sengupta et al. 2011 |
| BASP1 | PubMed | Antoniali et al. 2017 |
| CAPNS1 | BioGRID | Kristensen, Gsponer, and Foster 2012 |
| CCDC124 | BioGRID | Kristensen, Gsponer, and Foster 2012 |
| CDK5 | PubMed | Antoniali et al. 2017 |
| CDKN1A | BIND | Hanson, Kim, and Deppert 2005 |
| CSNK2A1 | HPRD | Fritz and Kaina 1999 |
| DDB1 | BioGRID | Wan et al. 2015 |
| ELOC | BioGRID | Kristensen, Gsponer, and Foster 2012 |
| EP300 | HPRD, BioGRID | Bhakat et al. 2003; Karmakar et al. 2010; Sengupta et al. 2011; Yamamori et al. 2010 |
| ESR1 | PubMed | Antoniali et al. 2017 |
| FEN1 | HPRD | Dianova, Bohr, and Dianov 2001; Huttlin et al. 2015 |
| GZMA | HPRD, BioGRID | Fan, Beresford, Zhang, et al. 2003; Kim, Kim, and Seo 2013; Pinkoski and Green 2003 |
| GZMK | BioGRID | Zhao et al. 2007 |
| HDAC1 | HPRD | Bhakat et al. 2003; Zhao et al. 2007 |
| HDAC2 | BioGRID | Bhakat et al. 2003 |
| HHV8GK18_gp81 | BioGRID | Shamay et al. 2012 |
| HIF1A | HPRD, BioGRID | Carrero et al. 2000 |
| HMGA1 | BioGRID | Sgarra et al. 2008 |
| HMGA2 | BioGRID | Sgarra et al. 2008 |
| HMGB2 | HPRD | Fan, Beresford, Zhang, et al. 2003 |
| HNRNPH1 | PubMed | Antoniali et al. 2017 |
| HNRNPK | BioGRID | Kristensen, Gsponer, and Foster 2012 |
| HNRNPL | HPRD, BioGRID | Kuninger et al. 2002 |
| HNRNPUL1 | BioGRID | Kristensen, Gsponer, and Foster 2012 |
| HOXC9 | PubMed | Antoniali et al. 2017 |
| HSPA1A | HPRD | Kenny et al. 2001; Mendez et al. 2000 |
| HSPA1B | PubMed | Antoniali et al. 2017 |
| HUS1 | PubMed | Antoniali et al. 2017 |
| KIF11 | PubMed | Antoniali et al. 2017 |
| KRT8 | BioGRID | Vascotto et al. 2009 |
| LGALS1 | BioGRID | Kristensen, Gsponer, and Foster 2012 |
| LIG1 | PubMed | Antoniali et al. 2017 |
| MDM2 | BioGRID | Busso, Iwakuma, and Izumi 2009 |
| MPG | PubMed | Antoniali et al. 2017 |
| MSN | PubMed | Antoniali et al. 2017 |
| MUTYH | BIND, HPRD, BioGRID | Luncsford et al. 2013; Parker et al. 2001 |
| MYH9 | PubMed | Antoniali et al. 2017 |
| MYO1C | PubMed | Antoniali et al. 2017 |
| NAE1 | BioGRID | Kristensen, Gsponer, and Foster 2012 |
| NME1 | HPRD | Fan, Beresford, Zhang, et al. 2003 |
| NPM1 | BioGRID | Vascotto et al. 2009 |
| NUDT3 | BioGRID | Vinayagam et al. 2011 |
| OGG1 | PubMed | Antoniali et al. 2017 |
| PABPC1 | BioGRID | Kristensen, Gsponer, and Foster 2012 |
| PCNA | HPRD, BioGRID | Dianova, Bohr, and Dianov 2001; Matsumoto et al. 1999 |
| POLB | HPRD, BioGRID | Bennett et al. 1997 |
| POLR3D | BioGRID | Ravasi et al. 2010 |
| PRDX6 | BioGRID | Vascotto et al. 2009 |
| PRKDC | BioGRID | Han et al. 2017 |
| PRPF19 | BioGRID | Vascotto et al. 2009 |
| PSMG1 | BioGRID | Kristensen, Gsponer, and Foster 2012 |
| RAD1 | PubMed | Antoniali et al. 2017 |
| RAD9A | PubMed | Antoniali et al. 2017 |
| REV | BioGRID | Naji et al. 2012 |
| RIC8A | BioGRID | Kristensen, Gsponer, and Foster 2012 |
| RNF4 | BioGRID | Hu et al. 2010 |
| RPA1 | BioGRID | Maréchal et al. 2014 |
| RPL3 | PubMed | Antoniali et al. 2017 |
| RPL14 | PubMed | Antoniali et al. 2017 |
| RPSA | BioGRID | Vascotto et al. 2009 |
| SET | HPRD, BioGRID | Fan, Beresford, Oh, et al. 2003; Fan, Beresford, Zhang, et al. 2003 |
| SFPQ | BioGRID | Havugimana et al. 2012 |
| SIRT1 | BioGRID | Yamamori et al. 2010 |
| SNRPD1 | BioGRID | Havugimana et al. 2012 |
| SPHK2 | PubMed | Antoniali et al. 2017 |
| SRPK1 | BioGRID | Varjosalo et al. 2013 |
| SRPK2 | BioGRID | Varjosalo et al. 2013 |
| STAT3 | BioGRID | Gray et al. 2005 |
| SUMO1 | PubMed | Antoniali et al. 2017 |
| SUMO2 | PubMed | Antoniali et al. 2017 |
| TCF21 | BioGRID | Ravasi et al. 2010 |
| TCP1 | BioGRID | Vascotto et al. 2009 |
| TDG | PubMed | Antoniali et al. 2017 |
| TERF1 | BioGRID | Lee et al. 2011 |
| TERF2 | BioGRID | Lee et al. 2011 |
| TERF2IP | BioGRID | Lee et al. 2011 |
| TFAP4 | BioGRID | Ku et al. 2009 |
| THRAP3 | PubMed | Antoniali et al. 2017 |
| TP53 | BIND, HPRD, BioGRID | Jayaraman et al. 1997; Seemann and Hainaut 2005 |
| TRAF2 | BioGRID | Merluzzi et al. 2008 |
| TWF2 | BioGRID | Kristensen, Gsponer, and Foster 2012 |
| TXN | HPRD, BioGRID | Hirota et al. 1997; Kristensen, Gsponer, and Foster 2012; Ueno et al. 1999; Wei et al. 2000 |
| TXNRD1 | BioGRID | Kristensen, Gsponer, and Foster 2012; Seemann and Hainaut 2005 |
| UBC | PubMed | Antoniali et al. 2017 |
| UBE2I | HPRD, BioGRID | Yan et al. 2000 |
| WDR77 | BioGRID | Vascotto et al. 2009 |
| XPOT | BioGRID | Kristensen, Gsponer, and Foster 2012 |
| XRCC1 | HPRD, BioGRID | Vidal et al. 2001; Yamamori et al. 2010 |
| XRCC5 | BioGRID | Chung et al. 1996 |
| XRCC6 | HPRD | Chung et al. 1996 |
| YBX1 | PubMed | Antoniali et al. 2017 |

**REFERENCES**

1. Antoniali, G. et al. Mammalian APE1 controls miRNA processing and its interactome is linked to cancer RNA metabolism. Nat Commun 8, 797 (2017).
2. Bennett, R. A. O., Wilson, D. M., Wong, D. & Demple, B. Interaction of human apurinic endonuclease and DNA polymerase in the base excision repair pathway. Proc. Natl. Acad. Sci. 94, 7166–7169 (1997).
3. Bhakat, K. K., Izumi, T., Yang, S.-H., Hazra, T. K. & Mitra, S. Role of acetylated human AP-endonuclease (APE1/Ref-1) in regulation of the parathyroid hormone gene. EMBO J. 22, 6299–6309 (2003).
4. Busso, C. S., Iwakuma, T. & Izumi, T. Ubiquitination of mammalian AP endonuclease (APE1) regulated by the p53–MDM2 signaling pathway. Oncogene 28, 1616–1625 (2009).
5. Carrero, P. et al. Redox-regulated recruitment of the transcriptional coactivators CREB-binding protein and SRC-1 to hypoxia-inducible factor 1alpha. Mol. Cell. Biol. 20, 402–15 (2000).
6. Chung, U. et al. The Interaction between Ku Antigen and REF1 Protein Mediates Negative Gene Regulation by Extracellular Calcium. J. Biol. Chem. 271, 8593–8598 (1996).
7. Dianova, I. I., Bohr, V. A. & Dianov, G. L. Interaction of Human AP Endonuclease 1 with Flap Endonuclease 1 and Proliferating Cell Nuclear Antigen Involved in Long-Patch Base Excision Repair. Biochemistry 40, 12639–12644 (2001).
8. Fan, Z. et al. Cleaving the oxidative repair protein Ape1 enhances cell death mediated by granzyme A. Nat. Immunol. 4, 145–153 (2003).
9. Fan, Z., Beresford, P. J., Oh, D. Y., Zhang, D. & Lieberman, J. Tumor suppressor NM23-H1 is a granzyme A-activated DNase during CTL-mediated apoptosis, and the nucleosome assembly protein SET is its inhibitor. Cell 112, 659–72 (2003).
10. Fritz, G. & Kaina, B. Phosphorylation of the DNA repair protein APE/REF-1 by CKII affects redox regulation of AP-1. Oncogene 18, 1033–1040 (1999).
11. Gray, M. J. et al. HIF-1α, STAT3, CBP/p300 and Ref-1/APE are components of a transcriptional complex that regulates Src-dependent hypoxia-induced expression of VEGF in pancreatic and prostate carcinomas. Oncogene 24, 3110–3120 (2005).
12. Han, K. et al. Synergistic drug combinations for cancer identified in a CRISPR screen for pairwise genetic interactions. Nat. Biotechnol. 35, 463–474 (2017).
13. Hanson, S., Kim, E. & Deppert, W. Redox factor 1 (Ref-1) enhances specific DNA binding of p53 by promoting p53 tetramerization. Oncogene 24, 1641–1647 (2005).
14. Havugimana, P. C. et al. A Census of Human Soluble Protein Complexes. Cell 150, 1068–1081 (2012).
15. Hirota, K. et al. AP-1 transcriptional activity is regulated by a direct association between thioredoxin and Ref-1. Proc. Natl. Acad. Sci. 94, 3633–3638 (1997).
16. Hu, X. V. et al. Identification of RING finger protein 4 (RNF4) as a modulator of DNA demethylation through a functional genomics screen. Proc. Natl. Acad. Sci. 107, 15087–15092 (2010).
17. Huttlin, E. L. et al. The BioPlex Network: A Systematic Exploration of the Human Interactome. Cell 162, 425–440 (2015).
18. Jayaraman, L. et al. Identification of redox/repair protein Ref-1 as a potent activator of p53. Genes Dev. 11, 558–570 (1997).
19. Karmakar, S., Mahajan, M. C., Schulz, V., Boyapaty, G. & Weissman, S. M. A multiprotein complex necessary for both transcription and DNA replication at the β-globin locus. EMBO J. 29, 3260–71 (2010).
20. Kenny, M. K. et al. Heat shock protein 70 binds to human apurinic/apyrimidinic endonuclease and stimulates endonuclease activity at abasic sites. J. Biol. Chem. 276, 9532–6 (2001).
21. Kim, H. L., Kim, S. U. & Seo, Y. R. A novel role for Gadd45α in base excision repair: Modulation of APE1 activity by the direct interaction of Gadd45α with PCNA. Biochem. Biophys. Res. Commun. 434, 185–190 (2013).
22. Kristensen, A. R., Gsponer, J. & Foster, L. J. A high-throughput approach for measuring temporal changes in the interactome. Nat. Methods 9, 907–909 (2012).
23. Ku, W.-C. et al. Complementary Quantitative Proteomics Reveals that Transcription Factor AP-4 Mediates E-box-dependent Complex Formation for Transcriptional Repression of HDM2. Mol. Cell. Proteomics 8, 2034–2050 (2009).
24. Kuninger, D. T., Izumi, T., Papaconstantinou, J. & Mitra, S. Human AP-endonuclease 1 and hnRNP-L interact with a nCaRE-like repressor element in the AP-endonuclease 1 promoter. Nucleic Acids Res. 30, 823–829 (2002).
25. Lee, O.-H. et al. Genome-wide YFP Fluorescence Complementation Screen Identifies New Regulators for Telomere Signaling in Human Cells. Mol. Cell. Proteomics 10, M110.001628 (2011).
26. Luncsford, P. J. et al. Coordination of MYH DNA glycosylase and APE1 endonuclease activities via physical interactions. DNA Repair (Amst). 12, 1043–52 (2013).
27. Maréchal, A. et al. PRP19 Transforms into a Sensor of RPA-ssDNA after DNA Damage and Drives ATR Activation via a Ubiquitin-Mediated Circuitry. Mol. Cell 53, 235–246 (2014).
28. Matsumoto, Y. et al. Reconstitution of proliferating cell nuclear antigen-dependent repair of apurinic/apyrimidinic sites with purified human proteins. J. Biol. Chem. 274, 33703–8 (1999).
29. Mendez, F. et al. Heat-shock proteins associated with base excision repair enzymes in HeLa cells. Radiat. Res. 153, 186–95 (2000).
30. Merluzzi, S., D’Orlando, O., Leonardi, A., Vitale, G. & Pucillo, C. TRAF2 and p38 are involved in B cells CD40-mediated APE/Ref-1 nuclear translocation: A novel pathway in B cell activation. Mol. Immunol. 45, 76–86 (2008).
31. Naji, S. et al. Host Cell Interactome of HIV-1 Rev Includes RNA Helicases Involved in Multiple Facets of Virus Production. Mol. Cell. Proteomics 11, M111.015313 (2012).
32. Oláh, J. et al. Interactions of Pathological Hallmark Proteins. J. Biol. Chem. 286, 34088–34100 (2011).
33. Parker, A. et al. Human Homolog of the MutY Repair Protein (hMYH) Physically Interacts with Proteins Involved in Long Patch DNA Base Excision Repair. J. Biol. Chem. 276, 5547–5555 (2001).
34. Pinkoski, M. J. & Green, D. R. Granzyme A: the road less traveled. Nat. Immunol. 4, 106–8 (2003).
35. Ravasi, T. et al. An Atlas of Combinatorial Transcriptional Regulation in Mouse and Man. Cell 140, 744–752 (2010).
36. Seemann, S. & Hainaut, P. Roles of thioredoxin reductase 1 and APE/Ref-1 in the control of basal p53 stability and activity. Oncogene 24, 3853–3863 (2005).
37. Sengupta, S., Mantha, A. K., Mitra, S. & Bhakat, K. K. Human AP endonuclease (APE1/Ref-1) and its acetylation regulate YB-1-p300 recruitment and RNA polymerase II loading in the drug-induced activation of multidrug resistance gene MDR1. Oncogene 30, 482–93 (2011).
38. Sgarra, R. et al. Interaction proteomics of the HMGA chromatin architectural factors. Proteomics 8, 4721–4732 (2008).
39. Shamay, M. et al. A Protein Array Screen for Kaposi’s Sarcoma-Associated Herpesvirus LANA Interactors Links LANA to TIP60, PP2A Activity, and Telomere Shortening. J. Virol. 86, 5179–5191 (2012).
40. Ueno, M. et al. Thioredoxin-dependent redox regulation of p53-mediated p21 activation. J. Biol. Chem. 274, 35809–15 (1999).
41. Varjosalo, M. et al. The Protein Interaction Landscape of the Human CMGC Kinase Group. Cell Rep. 3, 1306–1320 (2013).
42. Vascotto, C. et al. APE1/Ref-1 Interacts with NPM1 within Nucleoli and Plays a Role in the rRNA Quality Control Process. Mol. Cell. Biol. 29, 1834–1854 (2009).
43. Vidal, A. E., Boiteux, S., Hickson, I. D. & Radicella, J. P. XRCC1 coordinates the initial and late stages of DNA abasic site repair through protein-protein interactions. EMBO J. 20, 6530–6539 (2001).
44. Vinayagam, A. et al. A Directed Protein Interaction Network for Investigating Intracellular Signal Transduction. Sci. Signal. 4, rs8–rs8 (2011).
45. Wan, C. et al. Panorama of ancient metazoan macromolecular complexes. Nature 525, 339–344 (2015).
46. Wei, S. J. et al. Thioredoxin nuclear translocation and interaction with redox factor-1 activates the activator protein-1 transcription factor in response to ionizing radiation. Cancer Res. 60, 6688–95 (2000).
47. Yamamori, T. et al. SIRT1 deacetylates APE1 and regulates cellular base excision repair. Nucleic Acids Res. 38, 832–845 (2010).
48. Yan, M.-D. et al. Ubiquitin Conjugating Enzyme Ubc9 is Involved in Protein Degradation of Redox Factor-1 (Ref-1). Sheng Wu Hua Xue Yu Sheng Wu Wu Li Xue Bao (Shanghai). 32, 63–68 (2000).
49. Zhao, T. et al. Granzyme K cleaves the nucleosome assembly protein SET to induce single-stranded DNA nicks of target cells. Cell Death Differ. 14, 489–499 (2007).
