# Supplementary material for: Architecture of The Human Ape1 Interactome Defines Novel Cancers Signatures
Source: Sci Rep. 2020 Jan 8;10:28. doi: 10.1038/s41598-019-56981-z (PMC6949240; doi:10.1038/s41598-019-56981-z)
Supplement: Supplementary file 7 — Supplementary Table S7. [file 41598_2019_56981_MOESM7_ESM.docx]

**Title of the Paper: ARCHITECTURE OF THE HUMAN APE1 INTERACTOME DEFINES NOVEL CANCERS SIGNATURES**

Authors list: Dilara Ayyildiz^1,#^, Giulia Antoniali^1,#^, Chiara D’Ambrosio^2,#^, Giovanna Mangiapane^1^, Emiliano Dalla^1^,Andrea Scaloni^2^, Gianluca Tell^1,^* and Silvano Piazza^3,^*

^1^Laboratory of Molecular Biology and DNA repair, Department of Medicine, University of Udine, p.le M. Kolbe 4, 33100 Udine, Italy; Phone: +39 0432 494311.

^2^Proteomics and Mass Spectrometry Laboratory, Institute for the Animal Production System in the Mediterranean Environment (ISPAAM), National Research Council (CNR) of Italy, via Argine 1085, 80147 Naples, Italy; Phone +39 081 5966006.

^3^Bioinformatics Core Facility, Centre for Integrative Biology (CIBIO), University of Trento, via Sommarive 18, 38123, Povo (Trento), Italy; Phone: +39 0461 283790.

^#^ These authors equally contributed to the present work.

***Corresponding authors**: Silvano Piazza (silvano.piazza@unitn.it) and Gianluca Tell (gianluca.tell@uniud.it)

**Supplementary Table S7.**

**APE1-PPI bad prognostic signatures top regulators analysis.**

GeneXplain identification of the Top 3 putative master regulators of bad prognostic genes in the 11 selected TCGA cancer datasets (ranked by ascending Ranks sum). The reference is given for the association with the proliferation, apoptosis and resistance functional terms, indicating the involvement of top upstream regulators in these pathways (x indicates that no reference was found).

| **Dataset** | **Top1** | **Gene Symbol Description** | **Proliferation** | **Apoptosis** | **Resistance** |
| --- | --- | --- | --- | --- | --- |
| **HNSC** | XRCC6 | X-ray repair cross-complementing protein 6 | 1 | 2–4 | 2 |
| **KIRC** | PRKCD | Protein kinase C delta type | 5 | 6–8 | 8,9 |
| **LUAD** | PRKN | Parkin RBR E3 Ubiquitin Protein Ligase | x | 10–12 | x |
| **UVM** | SMYD2 | SET And MYND Domain Containing 2 | 13–15 | 16,17 | 17 |
| **LGG** | SETD7 | SET Domain Containing 7, Histone Lysine Methyltransferase | 18,19 | 19,20 | 21 |
| **SKCM** | DDB1 | Damage Specific DNA Binding Protein 1 | 22,23 | 24,25 | 22 |
| **LIHC** | PRKN | Parkin RBR E3 Ubiquitin Protein Ligase | x | 10–12 | x |
| **KIRP** | YBX1 | Y-Box Binding Protein 1 | 26–29 | 26–28 | 27,28 |
| **BRCA** | DDB1 | Damage Specific DNA Binding Protein 1 | 22,23 | 24,25 | 22 |
| **BLCA** | DDB1 | Damage Specific DNA Binding Protein 1 | 22,23 | 24,25 | 22 |
| **PAAD** | YBX1 | Y-Box Binding Protein 1 | 26–29 | 26–28 | 27,28 |
| **Dataset** | **Top2** | **Gene Symbol Description** | **Proliferation** | **Apoptosis** | **Resistance** |
| **HNSC** | PRKDC | Protein Kinase, DNA-Activated, Catalytic Subunit | 30–32 | 30,31,33 | 33,34 |
| **KIRC** | PPP1CB | Protein Phosphatase 1 Catalytic Subunit Beta | x | 35 | x |
| **LUAD** | HDAC2 | Histone deacetylase 2 | 36–38 | 36,39 | 37,40 |
| **UVM** | KDM1A | Lysine Demethylase 1A | 41 | 41–44 | 44,45 |
| **LGG** | KDM1A | Lysine Demethylase 1A | 41 | 41–44 | 4,45 |
| **SKCM** | PTP4A3 | Protein Tyrosine Phosphatase 4A3 | 46–49 | 46,48 | 46 |
| **LIHC** | HDAC2 | Histone deacetylase 2 | 36–38 | 36,39 | 37,40 |
| **KIRP** | PTP4A3 | Protein Tyrosine Phosphatase 4A3 | 46–49 | 46,48 | 46 |
| **BRCA** | XRCC6 | X-ray repair cross-complementing protein 6 | 1 | 2–4 | 2 |
| **BLCA** | XRCC5 | X-ray repair cross-complementing protein 5 | 50 | 4,51 | x |
| **PAAD** | ACTL6A | Actin-like protein 6A | 52,53 | 54 | x |
| Dataset | **Top3** | Gene Symbol Description | Proliferation | Apoptosis | Resistance |
| **HNSC** | TERF2 | Telomeric Repeat Binding Factor 2 | x | 55 | 56 |
| **KIRC** | YBX1 | Y-Box Binding Protein 1 | 26–29 | 26–28 | 27,28 |
| **LUAD** | XRCC6 | X-ray repair cross-complementing protein 6 | 1 | 2–4 | 2 |
| **UVM** | XRCC5 | X-ray repair cross-complementing protein 5 | 50 | 4,51 | x |
| **LGG** | CUL1 | Cullin-1 | 57,58 | 59 | x |
| **SKCM** | XRCC6 | X-ray repair cross-complementing protein 6 | 1 | 2–4 | 2 |
| **LIHC** | XRCC6 | X-ray repair cross-complementing protein 6 | 1 | 2–4 | 2 |
| **KIRP** | TCF21 | Transcription factor 21 | 60,61 | 60,61 | 61,62 |
| **BRCA** | IDE | Insulin-degrading enzyme | 63 | 64 | x |
| **BLCA** | TRIM28 | Tripartite Motif Containing 28 | 65–67 | 65–67 | 66 |
| **PAAD** | HNRNPK | Heterogeneous nuclear ribonucleoprotein K | 68–70 | 68,71,72 | 73 |

**REFERENCES**

1. Zhu, B., Cheng, D., Li, S., Zhou, S. & Yang, Q. High Expression of XRCC6 Promotes Human Osteosarcoma Cell Proliferation through the β-Catenin/Wnt Signaling Pathway and Is Associated with Poor Prognosis. *Int. J. Mol. Sci.* **17**, 1188 (2016).

2. Pavón, M. A. *et al.* *Ku* 70 predicts response and primary tumor recurrence after therapy in locally advanced head and neck cancer. *Int. J. Cancer* **123**, 1068–1079 (2008).

3. Ren, F. *et al.* DNA-PKcs and Ku70 are Predictive Markers for Poor Prognosis of Patients With Gall Bladder Malignancies. *Appl. Immunohistochem. Mol. Morphol.* **22**, 741–747 (2014).

4. Bau, D.-T., Tsai, C.-W. & Wu, C.-N. Role of the XRCC5/XRCC6 dimer in carcinogenesis and pharmacogenomics. *Pharmacogenomics* **12**, 515–534 (2011).

5. Yang, Q.-E., Ozawa, M., Zhang, K., Johnson, S. E. & Ealy, A. D. The requirement for protein kinase C delta (PRKCD) during preimplantation bovine embryo development. *Reprod. Fertil. Dev.* **28**, 482 (2016).

6. Baumann, U. *et al.* Disruption of the PRKCD–FBXO25–HAX-1 axis attenuates the apoptotic response and drives lymphomagenesis. *Nat. Med.* **20**, 1401–1409 (2014).

7. Geraldes, P. *et al.* Activation of PKC-δ and SHP-1 by hyperglycemia causes vascular cell apoptosis and diabetic retinopathy. *Nat. Med.* **15**, 1298–1306 (2009).

8. Ke, G. *et al.* MiR-181a confers resistance of cervical cancer to radiation therapy through targeting the pro-apoptotic PRKCD gene. *Oncogene* **32**, 3019–3027 (2013).

9. Chen, Y. *et al.* MicroRNA-181a enhances the chemoresistance of human cervical squamous cell carcinoma to cisplatin by targeting PRKCD. *Exp. Cell Res.* **320**, 12–20 (2014).

10. Zhou, Y. *et al.* Topology-dependent, bifurcated mitochondrial quality control under starvation. *Autophagy* 1–13 (2019). doi:10.1080/15548627.2019.1634944

11. Xian, H. & Liou, Y.-C. Loss of MIEF1/MiD51 confers susceptibility to BAX-mediated cell death and PINK1-PRKN-dependent mitophagy. *Autophagy* 1–19 (2019). doi:10.1080/15548627.2019.1596494

12. Guida, M. *et al.* Parkin Interacts with Apoptosis-Inducing Factor and Interferes with Its Translocation to the Nucleus in Neuronal Cells. *Int. J. Mol. Sci.* **20**, 748 (2019).

13. Ohtomo-Oda, R. *et al.* SMYD2 overexpression is associated with tumor cell proliferation and a worse outcome in human papillomavirus–unrelated nonmultiple head and neck carcinomas. *Hum. Pathol.* **49**, 145–155 (2016).

14. Thomenius, M. J. *et al.* Small molecule inhibitors and CRISPR/Cas9 mutagenesis demonstrate that SMYD2 and SMYD3 activity are dispensable for autonomous cancer cell proliferation. *PLoS One* **13**, e0197372 (2018).

15. Zipin-Roitman, A. *et al.* SMYD2 lysine methyltransferase regulates leukemia cell growth and regeneration after genotoxic stress. *Oncotarget* **8**, 16712–16727 (2017).

16. Sajjad, A. *et al.* Lysine methyltransferase Smyd2 suppresses p53-dependent cardiomyocyte apoptosis. *Biochim. Biophys. Acta - Mol. Cell Res.* **1843**, 2556–2562 (2014).

17. Shang, L. & Wei, M. Inhibition of SMYD2 Sensitized Cisplatin to Resistant Cells in NSCLC Through Activating p53 Pathway. *Front. Oncol.* **9**, (2019).

18. Chen, Y. *et al.* Increased Expression of SETD7 Promotes Cell Proliferation by Regulating Cell Cycle and Indicates Poor Prognosis in Hepatocellular Carcinoma. *PLoS One* **11**, e0154939 (2016).

19. Duan, B. *et al.* Histone-lysine N-methyltransferase SETD7 is a potential serum biomarker for colorectal cancer patients. *EBioMedicine* **37**, 134–143 (2018).

20. Huang, R. *et al.* SETD7 is a prognosis predicting factor of breast cancer and regulates redox homeostasis. *Oncotarget* **8**, 94080–94090 (2017).

21. Oudhoff, M. J. *et al.* Intestinal Epithelial Cell-Intrinsic Deletion of Setd7 Identifies Role for Developmental Pathways in Immunity to Helminth Infection. *PLOS Pathog.* **12**, e1005876 (2016).

22. Leung-Pineda, V., Huh, J. & Piwnica-Worms, H. DDB1 Targets Chk1 to the Cul4 E3 Ligase Complex in Normal Cycling Cells and in Cells Experiencing Replication Stress. *Cancer Res.* **69**, 2630–2637 (2009).

23. Yamaji, S. *et al.* Hepatocyte-specific deletion of DDB1 induces liver regeneration and tumorigenesis. *Proc. Natl. Acad. Sci.* **107**, 22237–22242 (2010).

24. Zhao, R. *et al.* DNA Damage-Binding Complex Recruits HDAC1 to Repress Bcl-2 Transcription in Human Ovarian Cancer Cells. *Mol. Cancer Res.* **12**, 370–380 (2014).

25. Cang, Y. *et al.* Deletion of DDB1 in Mouse Brain and Lens Leads to p53-Dependent Elimination of Proliferating Cells. *Cell* **127**, 929–940 (2006).

26. Zhou, L. *et al.* High YBX1 expression indicates poor prognosis and promotes cell migration and invasion in nasopharyngeal carcinoma. *Exp. Cell Res.* **361**, 126–134 (2017).

27. Liang, C. *et al.* Y-box binding protein-1 promotes tumorigenesis and progression via the epidermal growth factor receptor/AKT pathway in spinal chordoma. *Cancer Sci.* **110**, 166–179 (2019).

28. Zhao, S. *et al.* YBX1 regulates tumor growth via CDC25a pathway in human lung adenocarcinoma. *Oncotarget* **7**, 82139–82157 (2016).

29. Kuwano, M., Shibata, T., Watari, K. & Ono, M. Oncogenic Y‐box binding protein‐1 as an effective therapeutic target in drug‐resistant cancer. *Cancer Sci.* **110**, 1536–1543 (2019).

30. Gurley, K. E., Ashley, A. K., Moser, R. D. & Kemp, C. J. Synergy between Prkdc and Trp53 regulates stem cell proliferation and GI-ARS after irradiation. *Cell Death Differ.* **24**, 1853–1860 (2017).

31. Gladdy, R. A., Nutter, L. M. J., Kunath, T., Danska, J. S. & Guidos, C. J. p53-Independent Apoptosis Disrupts Early Organogenesis in Embryos Lacking Both Ataxia-Telangiectasia Mutated and Prkdc. *Mol. Cancer Res.* **4**, 311–318 (2006).

32. Habiel, D. M. *et al.* DNA-PKcs modulates progenitor cell proliferation and fibroblast senescence in idiopathic pulmonary fibrosis. *BMC Pulm. Med.* **19**, 165 (2019).

33. Mori, N., Matsumoto, Y., Okumoto, M., Suzuki, N. & Yamate, J. Variations in Prkdc encoding the catalytic subunit of DNA-dependent protein kinase (DNA-PKcs) and susceptibility to radiation-induced apoptosis and lymphomagenesis. *Oncogene* **20**, 3609–3619 (2001).

34. Sun, G. *et al.* PRKDC regulates chemosensitivity and is a potential prognostic and predictive marker of response to adjuvant chemotherapy in breast cancer patients. *Oncol. Rep.* **37**, 3536–3542 (2017).

35. Madsen, C. D. *et al.* STRIPAK components determine mode of cancer cell migration and metastasis. *Nat. Cell Biol.* **17**, 68–80 (2015).

36. Li, S. *et al.* HDAC2 regulates cell proliferation, cell cycle progression and cell apoptosis in esophageal squamous cell carcinoma EC9706 cells. *Oncol. Lett.* **13**, 403–409 (2017).

37. Zhang, Z. *et al.* Silencing of histone deacetylase 2 suppresses malignancy for proliferation, migration, and invasion of glioblastoma cells and enhances temozolomide sensitivity. *Cancer Chemother. Pharmacol.* **78**, 1289–1296 (2016).

38. Jurkin, J. *et al.* Distinct and redundant functions of histone deacetylases HDAC1 and HDAC2 in proliferation and tumorigenesis. *Cell Cycle* **10**, 406–412 (2011).

39. Lin, C.-L. *et al.* HDAC1 and HDAC2 Double Knockout Triggers Cell Apoptosis in Advanced Thyroid Cancer. *Int. J. Mol. Sci.* **20**, 454 (2019).

40. Zhao, H. *et al.* HDAC2 overexpression is a poor prognostic factor of breast cancer patients with increased multidrug resistance-associated protein expression who received anthracyclines therapy. *Jpn. J. Clin. Oncol.* **46**, 893–902 (2016).

41. Huang, Y., Zou, Y., Lin, L., Ma, X. & Zheng, R. miR-101 regulates cell proliferation and apoptosis by targeting KDM1A in diffuse large B cell lymphoma. *Cancer Manag. Res.* **Volume 11**, 2739–2746 (2019).

42. Sareddy, G. R. *et al.* Novel KDM1A inhibitors induce differentiation and apoptosis of glioma stem cells via unfolded protein response pathway. *Oncogene* **36**, 2423–2434 (2017).

43. Cai, L., Chen, Q., Fang, S., Lian, M. & Cai, M. MicroRNA-329 inhibits cell proliferation and tumor growth while facilitates apoptosis via negative regulation of KDM1A in gastric cancer. *J. Cell. Biochem.* **119**, 3338–3351 (2018).

44. Huang, M. *et al.* Targeting KDM1A attenuates Wnt/β-catenin signaling pathway to eliminate sorafenib-resistant stem-like cells in hepatocellular carcinoma. *Cancer Lett.* **398**, 12–21 (2017).

45. Pishas, K. I. *et al.* Therapeutic Targeting of KDM1A/LSD1 in Ewing Sarcoma with SP-2509 Engages the Endoplasmic Reticulum Stress Response. *Mol. Cancer Ther.* **17**, 1902–1916 (2018).

46. den Hollander, P. *et al.* Phosphatase PTP4A3 Promotes Triple-Negative Breast Cancer Growth and Predicts Poor Patient Survival. *Cancer Res.* **76**, 1942–1953 (2016).

47. Sun, H.-Y. *et al.* Decreased expression of miR-551b predicts poor prognosis and promotes tumorigenesis by targeting PTP4A3 in human colorectal cancer. *Eur. Rev. Med. Pharmacol. Sci.* **23**, 5741–5751 (2019).

48. Vandsemb, E. N. *et al.* Phosphatase of regenerating liver 3 (PRL-3) is overexpressed in human prostate cancer tissue and promotes growth and migration. *J. Transl. Med.* **14**, 71 (2016).

49. Wang, L. *et al.* PTP4A3 is a target for inhibition of cell proliferatin, migration and invasion through Akt/mTOR signaling pathway in glioblastoma under the regulation of miR-137. *Brain Res.* **1646**, 441–450 (2016).

50. Xiao, Y. *et al.* Ku80 cooperates with CBP to promote COX-2 expression and tumor growth. *Oncotarget* **6**, (2015).

51. Kalra, R. & Bapat, S. A. Enhanced levels of double-strand DNA break repair proteins protect ovarian cancer cells against genotoxic stress-induced apoptosis. *J. Ovarian Res.* **6**, 66 (2013).

52. Meng, L. *et al.* BAF53a is a potential prognostic biomarker and promotes invasion and epithelial-mesenchymal transition of glioma cells. *Oncol. Rep.* **38**, 3327–3334 (2017).

53. Krasteva, V. *et al.* The BAF53a subunit of SWI/SNF-like BAF complexes is essential for hemopoietic stem cell function. *Blood* **120**, 4720–4732 (2012).

54. Choi, E. Y., Park, J. A., Sung, Y. H. & Kwon, H. Generation of the Dominant-Negative Mutant of hArpNβ: A Component of Human SWI/SNF Chromatin Remodeling Complex. *Exp. Cell Res.* **271**, 180–188 (2001).

55. Kim, J., Choi, I. & Lee, Y. Involvement of Atm and Trp53 in neural cell loss due to Terf2 inactivation during mouse brain development. *Histochem. Cell Biol.* **148**, 489–501 (2017).

56. Benhamou, Y. *et al.* Telomeric repeat-binding factor 2: a marker for survival and anti-EGFR efficacy in oral carcinoma. *Oncotarget* **7**, 44236–44251 (2016).

57. Cheng, Q. & Yin, G. Cullin-1 Regulates MG63 Cell Proliferation and Metastasis and is a Novel Prognostic Marker of Osteosarcoma. *Int. J. Biol. Markers* **32**, 202–209 (2017).

58. Ping, J.-G. *et al.* The expression of Cullin1 is increased in renal cell carcinoma and promotes cancer cell proliferation, migration, and invasion. *Tumor Biol.* **37**, 12823–12831 (2016).

59. jie, C., bai-yong, S., xia-xing, D., qian, Z. & cheng-hong, P. SKP1-CULLIN1-F-box (SCF)-mediated DRG2 degradation facilitated chemotherapeutic drugs induced apoptosis in hepatocellular carcinoma cells. *Biochem. Biophys. Res. Commun.* **420**, 651–655 (2012).

60. Dai, Y. *et al.* Down-regulation of TCF21 by hypermethylation induces cell proliferation, migration and invasion in colorectal cancer. *Biochem. Biophys. Res. Commun.* **469**, 430–436 (2016).

61. Yang, Z. *et al.* TCF21 inhibits proliferation and chemoresistance through the AKT pathway in human gastric cancer. *Gene* **682**, 42–49 (2019).

62. Akama, T. & Chun, T.-H. Transcription factor 21 (TCF21) promotes proinflammatory interleukin 6 expression and extracellular matrix remodeling in visceral adipose stem cells. *J. Biol. Chem.* **293**, 6603–6610 (2018).

63. Pivovarova, O. *et al.* Modulation of insulin degrading enzyme activity and liver cell proliferation. *Cell Cycle* **14**, 2293–2300 (2015).

64. Li, H. *et al.* cAMP/PKA signaling pathway contributes to neuronal apoptosis via regulating IDE expression in a mixed model of type 2 diabetes and Alzheimer’s disease. *J. Cell. Biochem.* **119**, 1616–1626 (2018).

65. Liu, L. *et al.* Downregulation of TRIM28 inhibits growth and increases apoptosis of nude mice with non‑small cell lung cancer xenografts. *Mol. Med. Rep.* **17**, 835–842 (2017).

66. Zhang, P.-P. *et al.* Expression of TRIM28 correlates with proliferation and Bortezomib-induced apoptosis in B-cell non-Hodgkin lymphoma. *Leuk. Lymphoma* **59**, 2639–2649 (2018).

67. Peng, Y., Zhang, M., Jiang, Z. & Jiang, Y. TRIM28 activates autophagy and promotes cell proliferation in glioblastoma. *Onco. Targets. Ther.* **Volume 12**, 397–404 (2019).

68. Chen, X. *et al.* Heterogeneous nuclear ribonucleoprotein K is associated with poor prognosis and regulates proliferation and apoptosis in bladder cancer. *J. Cell. Mol. Med.* **21**, 1266–1279 (2017).

69. Xu, Y. *et al.* The multifunctional RNA-binding protein hnRNPK is critical for the proliferation and differentiation of myoblasts. *BMB Rep.* **51**, 350–355 (2018).

70. Huang, H. *et al.* HNRNPK inhibits gastric cancer cell proliferation through p53/p21/CCND1 pathway. *Oncotarget* **8**, 103364–103374 (2017).

71. Huang, W. S. *et al.* ERK1/2-mediated Cytoplasmic Accumulation of hnRNPK Antagonizes TRAIL-induced Apoptosis through Upregulation of XIAP in H1299 Cells. *Biomed. Environ. Sci.* **30**, 473–481 (2017).

72. Yang, J.-H. *et al.* Arginine methylation of hnRNPK negatively modulates apoptosis upon DNA damage through local regulation of phosphorylation. *Nucleic Acids Res.* **42**, 9908–9924 (2014).

73. Daskalaki, W. *et al.* Expression levels of hnRNPK and p21WAF1/CIP1 are associated with resistance to radiochemotherapy independent of p53 pathway activation in rectal adenocarcinoma. *Int. J. Mol. Med.* **42**, 3269–3277 (2018).
