# Supplementary material for: Architecture of The Human Ape1 Interactome Defines Novel Cancers Signatures
Source: Sci Rep. 2020 Jan 8;10:28. doi: 10.1038/s41598-019-56981-z (PMC6949240; doi:10.1038/s41598-019-56981-z)

ARCHITECTURE OF THE HUMAN APE1 INTERACTOME DEFINES NOVEL CANCERS SIGNATURES

Dilara Ayyildiz[1,#], Giulia Antoniali[1,#], Chiara D'Ambrosio[2,#], Giovanna Mangiapane[1], Emiliano Dalla[1], Andrea Scaloni[2], Gianluca Tell[1,*] and Silvano Piazza[3,*]

[1]Laboratory of Molecular Biology and DNA repair, Department of Medicine, University of Udine, p.le M. Kolbe 4, 33100 Udine, Italy; Phone: +39 0432 494311.

[2]Proteomics and Mass Spectrometry Laboratory, Institute for the Animal Production System in the Mediterranean Environment (ISPAAM), National Research Council (CNR) of Italy, via Argine 1085, 80147 Naples, Italy; Phone +39 081 5966006.

[3]Bioinformatics Core Facility, Centre for Integrative Biology (CIBIO), University of Trento, via Sommarive 18, 38123, Povo (Trento), Italy; Phone: +39 0461 283790.

[#] These authors equally contributed to the present work.

***Corresponding authors**: Silvano Piazza (silvano.piazza@unitn.it) and Gianluca Tell (gianluca.tell@uniud.it)

SUPPLEMENTARY MATERIAL

List of contents:

- Pages S2 to S4, Supplementary Materials and Methods;
- Pages S5 to S9, Supplementary Figure Legends;
- Pages S10 to S12, Supplementary Table Legends;
- Pages S13, References.

Supplementary Materials and Methods

List of primary and secondary antibody used for western blot and PLA

Antigen	Company
APE1	Novus, NB 100-116
SFPQ	Abcam, ab38148
DHX9	Bethyl Laboratories, A300-854A
hnRNPK	Abcam, ab70492
hnRNPA2B1	Thermo Fisher Scientific , PA5-3439
LSD1	Abcam, ab129195
Tubulin	Sigma, T0198
NPM1	Abcam, ab15440
Anti-mouse IgG IRDye 800	LI-COR
Anti-rabbit IgG IRDye 680	LI-COR

Preparation of cell extracts and co-immunoprecipitation

Immunoprecipitation studies were carried out with whole cell extracts, and nuclear or cytoplasmic subfractions of HeLa cell clones[1,2]. For whole cell extracts, HeLa cell clones were seeded in two 150-cm plates at a density of 2 x 10^7 cells per plate. For nuclear and cytoplasmic fractions, HeLa cell clones were seeded in three 150-cm plates at a density of 2 x 10^7 cells per plate. For whole cell extracts cell, were lysed in lysis buffer containing 50 mM Tris-HCl pH7.4, 150 mM NaCl, 1mM EDTA and 1% Triton X-100. For nuclear and cytoplasmic fractions, cells were centrifuged at 800 × g for 10 min at 4 °C and the supernatant was removed. Pellet was re-suspended in a cold hypotonic solution containing 10 mM HEPES pH 7.9, 10 mM KCl, 0.1 mM $MgCl_2$, 0.1 mM EDTA pH 8.0 complemented with protease inhibitors. After centrifugation at 800 × g for 10 min at 4 °C, cytosolic proteins were collected whereas intact nuclei were pelleted. Pellet was washed to discard any contamination from cytosol and it was subsequently re-suspended with a cold hypertonic solution 20 mM HEPES pH 7.9, 400 mM NaCl, 1.5 mM $MgCl_2$, 0.1 mM EDTA pH 8.0, 5% glycerol complemented with protease inhibitors and incubated on ice for 30 min. At the end, the sample was centrifuged at 15,000 × g for 20 min at 4 °C and the supernatant containing nuclear proteins was collected.

Whole cell extracts, nuclear and cytoplasmic fractions were co-immunoprecipitated with anti-FLAG M2 affinity gel (Sigma-Aldrich) at 4 °C, with gentle rocking, for 3 h. After three washes with Tris-

buffered saline (TBS), immunoprecipitates were eluted through incubation with FLAG peptide (0.15 mg/ml) in TBS, and further characterized (see below). In parallel, immunoprecipitation of cell extracts from HeLa cells expressing APE1 FLAG-tagged was also performed with a resin lacking the FLAG antibody.

Immunofluorescence confocal and Proximity Ligation analyses

Immunofluorescence procedures and Proximity Ligation Assay (PLA) were carried out as described earlier [3]. To study the interaction between APE1 and three identified protein interactors *in vivo*, we used the *in situ* Proximity Ligation Assay technology (Duolink, Sigma-Aldrich). After incubation with monoclonal anti-APE1 (NB 100-116, Novus) (1:100) for 3 h, at 37 °C, cells were incubated with polyclonal anti-SFPQ (ab38148, Abcam, Cambridge, MA) (1:200), anti-DHX9 (A300-854A, Bethyl Laboratories, USA) (1:100), anti-hnRNPK (ab70492, Abcam) (1:200) and anti-hnRNPA2B1 (PA5-34939, Thermo Fisher Scientific, USA) (1:500), overnight, at 4 °C. PLA was performed following the manufacturer's instructions. Technical controls, represented by HeLa cell clones silenced for APE1 expression, resulted in the complete loss of PLA signal. Cells were visualized through a Leica TCS SP8 confocal system (Leica Microsystems GmbH, Germany).

Antibodies used and Western blotting analysis

For Western blotting analyses, cell lysates were resolved on 12% T SDS-PAGE, transferred onto nitrocellulose membranes (Amersham™ Protran™, GE Healthcare) and probed with antibodies for APE1 (NB 100-116, Novus) (1:1000), FLAG (F1804, SIGMA) (1:5000), LSD1 (ab129195, Abcam) (1:10000), β-tubulin (T0198, SIGMA) (1:2000) and NPM1 (ab15440, Abcam) (1:1000). The corresponding secondary antibodies labeled with IR-Dye (anti-rabbit IgG IRDye 680 and anti-mouse IgG IRDye 800) were used. Detection and quantification was performed with the Odyssey CLx Infrared imaging system (LI-COR GmbH, Germany). The membranes were scanned in two different channels using an Odyssey IR imager; protein bands were quantified using Odyssey software (Image Studio 5.0).

Proteomic analysis

Protein digests of gel slices from immunoprecipitated material of whole, nuclear and cytoplasmic cell extracts of HeLa cell clones expressing ectopic APE1 FLAG-tagged protein or stably transfected with the empty vector (SCR) were analyzed through Mass-Spectrometry. As a negative control, identical cell extracts from HeLa cells expressing APE1 FLAG-tagged were co-immunoprecipitated, in parallel, with a resin lacking the FLAG antibody (res). Mass-Spectrometry analyses were performed with a nanoLC-ESI-Q-Orbitrap-MS/MS platform consisting of an UltiMate 3000 HPLC RSLC nano system (Thermo Fisher Scientific, USA) coupled to a Q-ExactivePlus mass spectrometer through a Nanoflex ion source (Thermo Fisher Scientific). Peptides were loaded on an Acclaim PepMap™ RSLC C18 column (150 mm × 75 µm ID, 2 µm particles, 100 Å pore size) (Thermo Fisher Scientific) and eluted with a gradient of solvent B (19.92/80/0.08 v/v/v water/acetonitrile/formic acid) in solvent A (99.9/0.1 v/v water/formic acid), at a flow rate of 300 nl/min. The gradient of solvent B started at 3%, increased to 40% over 40 min, raised to 80% over 5 min, remained at 80% for 4 min, and finally returned to 3% in 1 min, with a column equilibrating step of 30 min before the subsequent chromatographic run. The mass spectrometer operated in data-dependent mode using a full scan (*m/z* range 375-1,500, a nominal resolution of 70,000, an automatic gain control target of 3,000,000, and a maximum ion target of 50 ms), followed by MS/MS scans of the 10 most abundant ions. MS/MS spectra were acquired in a scan *m/z* range 200-2000, using a normalized collision energy of 32%, an automatic gain control target of 100,000, a maximum ion target of 100 ms, and a resolution of 17,500. A dynamic exclusion value of 30s was also used. Duplicate analysis of each sample was performed to increase the number of identified peptides/protein coverage.

MS and MS/MS raw data files per lane were merged for protein identification into the Proteome Discoverer v. 2.1 software (Thermo Scientific), enabling the database search by the Mascot algorithm v. 2.4.2 (Matrix Science, UK) with the following parameters: UniProtKB human protein database (159,615 sequences) including the most common protein contaminants; carbamidomethylation of Cys as fixed modification; oxidation of Met, deamidation of Asn and Gln, and pyroglutamate formation of Gln as

variable modifications. Peptide mass tolerance and fragment mass tolerance were set to ± 10 ppm and ± 0.05 Da, respectively. Proteolytic enzyme and maximum number of missed cleavages were set to trypsin and 2, respectively. Protein candidates assigned on the basis of at least two sequenced peptides and Mascot score ≥30 were considered confidently identified. Definitive peptide assignment was always associated with manual spectra visualization and verification. Results were filtered to 1% false discovery rate. A comparison with results from the corresponding samples from control experiments (SCR and res) allowed to identify contaminant proteins in each experiment that, despite their abundance, were removed from the list of APE1-interacting partners (Supplementary Table S1 and S2).

Cell viability and proliferation

Cell viability was measured by using the 3-(4-5-dimethylthiazol-2-yl)-5-(3-carboxymethoxyphenyl)-2-(4-sulfophenyl)-2H-tetrazolium salt (MTS) assay (Celltiter 96 Aqueous One solution cell proliferation assay, Promega) on cells grown in 96-well plates and treated with Compound #3[4] and rotenone for 24 h. After treatment, the MTS solution was added to each well and the plates were incubated for 2 h, at 37 °C. Absorbance was measured at 490 nm by using a multi-well plate reader. The values were standardized to wells containing media alone.

Supplementary Figures

Supplementary Figure S1.

A) Interaction experiments on the APE1-binding proteins identified in this study. The PLA technology was used to evaluate *in vivo* the APE1-SFPQ, -DHX9 and -hnRNPK interaction. The PLA reaction was performed following manufacturer's instructions. Different scan areas and zoomed cells are presented relative to HeLa cell clones.

B) The PLA reaction was carried out using anti-APE1 and anti-SFPQ antibodies in A549 cell lines silenced for APE1 (siAPE1) or in relative control cell lines transfected with a scramble siRNA (SCR).

C) The PLA reaction was carried out using anti-APE1 and anti-hnRNPA2B1 antibodies in JHH-6 cell lines silenced for APE1 (siAPE1) or in relative control cell lines transfected with a scramble siRNA (SCR).

Supplementary Figure S2.

Interaction experiments on the APE1-binding proteins identified in this study. The PLA technology was used to evaluate *in vivo* the APE1-SFPQ, -DHX9 and -hnRNPK interaction. The PLA reaction was performed following manufacturer's instructions. HeLa cells clones silenced for the expression of APE1 were seeded on a glass coverslip and the PLA reaction was carried out using anti-APE1 and anti-SFPQ (A), anti-DHX9 (B) and anti-hnRNPK (C) antibodies. Confocal microscopy analysis highlighted the presence of distinct fluorescent red dots (PLA signals) indicating the occurrence of *in vivo* interaction between APE1 and its protein partners in HeLa cell clones expressing scramble siRNA (SCR). Few spots were visible in HeLa cell clones silenced for APE1 protein (siAPE1). DAPI staining was used as a reference for the nuclei. Bars, 10 µM. D) APE1 protein level evaluated in HeLa cells clones silenced for 10 days with doxycycline. SCR represents the control clone expressing the scramble shRNA; siRNA represents the clones silenced for APE1. Actin was used as loading control.

Supplementary Figure S3.

Venn diagram showing the common elements between the APE1-PPIs (n=531), the genes that were differentially expressed upon siAPE1 (n=836) and the genes whose transcripts were bound by APE1 in the RIP-seq experiment (n=913). Globally, APE1 regulates 95 unique genes at the transcriptional or post-transcriptional level in HeLa cells.

Supplementary Figure S4.

A) Bad Prognosis network of the HNSC dataset formed by the interactors of APE1. Symbolism and coloring of APE1, nodes and upstream regulators is identical to that shown in Fig 6.

B) Kaplan-Meier plot for the HNSC bad prognosis network.

C) Functional annotation of the HNSC bad prognosis network based on Gene Ontology - Biological Process terms (p<0.05). In the pie chart, the percentage of the genes enriched in the pathways are given next to the enriched terms.

Supplementary Figure S5.

A) Bad Prognosis network of the KIRC dataset formed by the interactors of APE1. Symbolism and coloring of APE1, nodes and upstream regulators is identical to that shown in Fig 6.

B) Kaplan-Meier plot for the KIRC bad prognosis network.

C) Functional annotation of the KIRC bad prognosis network based on Gene Ontology - Biological Process terms (p<0.05). In the pie chart, the percentage of the genes enriched in the pathways are given next to the enriched terms.

Supplementary Figure S6.

A) Bad Prognosis network of the UVM dataset formed by the interactors of APE1. Symbolism and coloring of APE1, nodes and upstream regulators is identical to that shown in Fig 6.

B) Kaplan-Meier plot for the UVM bad prognosis network.

C) Functional annotation of the UVM bad prognosis network based on Gene Ontology - Biological Process terms ($p<0.05$). In the pie chart, the percentage of the genes enriched in the pathways are given next to the enriched terms.

Supplementary Figure S7.

A) Bad Prognosis network of the LGG dataset formed by the interactors of APE1. Symbolism and coloring of APE1, nodes and upstream regulators is identical to that shown in Fig 6.

B) Kaplan-Meier plot for the LGG bad prognosis network.

C) Functional annotation of the LGG bad prognosis network based on Gene Ontology - Biological Process terms ($p<0.05$). In the pie chart, the percentage of the genes enriched in the pathways are given next to the enriched terms.

Supplementary Figure S8.

A) Bad Prognosis network of the SKCM dataset formed by the interactors of APE1. Symbolism and coloring of APE1, nodes and upstream regulators is identical to that shown in Fig 6.

B) Kaplan-Meier plot for the SKCM bad prognosis network.

C) Functional annotation of the SKCM bad prognosis network based on Gene Ontology - Biological Process terms ($p<0.05$). In the pie chart, the percentage of the genes enriched in the pathways are given next to the enriched terms.

Supplementary Figure S9.

A) Bad Prognosis network of the KIRP dataset formed by the interactors of APE1. Symbolism and coloring of APE1, nodes and upstream regulators is identical to that shown in Fig 6.

B) Kaplan-Meier plot for the KIRP bad prognosis network.

C) Functional annotation of the KIRP bad prognosis network based on Gene Ontology - Biological Process terms (p<0.05). In the pie chart, the percentage of the genes enriched in the pathways are given next to the enriched terms.

Supplementary Figure S10.

A) Bad Prognosis network of the BRCA dataset formed by the interactors of APE1. Symbolism and coloring of APE1, nodes and upstream regulators is identical to that shown in Fig 6.

B) Kaplan-Meier plot for the BRCA bad prognosis network.

C) Functional annotation of the BRCA bad prognosis network based on Gene Ontology - Biological Process terms (p<0.05). In the pie chart, the percentage of the genes enriched in the pathways are given next to the enriched terms.

Supplementary Figure S11.

A) Bad Prognosis network of the BLCA dataset formed by the interactors of APE1. Symbolism and coloring of APE1, nodes and upstream regulators is identical to that shown in Fig 6.

B) Kaplan-Meier plot for the BLCA bad prognosis network.

C) Functional annotation of the BLCA bad prognosis network based on Gene Ontology - Biological Process terms (p<0.05). In the pie chart, the percentage of the genes enriched in the pathways are given next to the enriched terms.

Supplementary Figure S12.

Dose-response plots of HeLa cells treated with Compound #3 and rotenone. HeLa cells were treated with increasing doses of these compounds. The MTS assay was used to quantify the relative levels of metabolic activity (A) and the Apo-ONE assay was used to quantify the relative levels of apoptosis (B). Data were normalized on untreated cells and represent the means ± SD of three independent experiments.

Asterisks represent a significant difference with respect to untreated cells. Data were evaluated statistically by two-tails Student t-test.

Supplementary Figure S13.

A) Uncropped blots for Figure 2.

B) Uncropped blots for Supplementary Figure S2C.

Supplementary Tables

Supplementary Table S1.

APE1-interacting proteins identified in this study. Reported are the rough identification results from co-immunoprecipitation experiments on whole cell lysate (WCE), and nuclear (NCE) or cytoplasmic (CCE) fractions of HeLa cell clones expressing the APE1 FLAG-tagged protein. Results were subtracted of identification data from the corresponding control experiments obtained with cell clones stably transfected with the empty vector (SCR) (also shown) or with HeLa cell clones expressing the APE1 FLAG-tagged protein treated with a resin lacking the FLAG antibody (RES) (also shown). The information on fraction (WCE, NCE or CCE), source sample (APE, SCR or RES), protein accession, description, gene, exp. *q*-value, sum PEP score, sequence coverage (%), number of identified peptides, PSMs, number of identified unique peptides, number of amino acids, molecular mass, pI, modification(s), identification confidence and Mascot identification score values is provided. Results are presented in different datasheets, showing in parallel the specific data for whole cell lysate (WCE), nuclear (NCE) or cytoplasmic (CCE) fractions, as well as the cumulative data from the combination of the three (ProteinIdentification_ALL). In each datasheet, results are grouped to show proteins uniquely present in the APE, SCR or RES samples, as well as those present in APE+SCR, APE+RES, SCR+RES or APE+SCR+RES; in each datasheet, these groups are indicated with a dedicated legend and are highlighted with different colors. Only proteins exclusively present in the APE group from whole cell lysate (WCE), nuclear (NCE) or cytoplasmic (CCE) fractions were used to define the APE1-PPI network (final n=455). This collection was then expanded with 80 additional components from previous interactomic investigations on this endonuclease [3] to generate the final list of proteins (n=535) used in the bioinformatic analysis.

Supplementary Table S2.

Identification details of the APE1-interacting proteins shown in Supplementary Table S1. Reported are the identified proteins (blue cells) and peptides (pink cells), as well as the corresponding identification

parameters. We show the information on fraction (WCE, NCE or CCE), protein false discovery rate (FDR) confidence, protein accession, description, gene, exp. *q*-value, sum PEP score, sequence coverage (%), number of identified peptides, PSMs, number of identified unique peptides, protein groups, number of amino acids, molecular mass, pI, Found in sample, modification(s), emPAI and Mascot identification score values. Specific information on the identified peptides for each protein are also provided, including identification confidence, sequence, modification, Quality PEP, Quality *q*-value, protein groups, proteins, PSMs, master protein, position, missed cleavage, theor. MH^+, Found in sample, ion score, Mascot confidence, Percolator PEP and Percolator *q*-values Mascot.

Supplementary Table S3.

The literature evidences for the APE1-interacting partners described in this study.

Supplementary Table S4.

Functional annotation terms of the global APE1-PPI network, APE1-PPI hubs network, and LUAD, LIHC and PAAD bad prognosis networks based on Gene Ontology - Biological Process terms (p<0.05) in sheet number 1, 2, 3, 4 and 5, respectively. Representative enrichment terms (lowest p-value) of each group that were later used in pie charts for the aforementioned datasets (See Figure 3 C & D and Figure 6 C, F &I) are highlighted. For abbreviations, see Supplementary Table S6.

Supplementary Table S5.

Identification of enriched transcription factor binding sites (TFBS) in the APE1 interactome gene promoters (-2500, -1nt from the TSS) using the LASAGNA-Search 2.0 tool. Worksheet "LASAGNA-Search-results_APE1_PPI" contains the raw results of the motif discovery analysis, showing for each promoter (n=531) the list of significantly enriched TFBS, sorted by increasing position within the FASTA formatted genomic sequence, as well as the matching sequence, strand, score, p-value and e-value. The worksheet "LASAGNA-APE1_PPI_Top10_pval" contains, for each promoter, the top10 most

informative hits, sorted by descending score and p-value (col. A-G). A list of unique transcription factors (TFs) binding these sites is shown in col. I, as well as a summary table indicating the overall number of promoters associated with every enriched TFBS, sorted by descending number of promoters (col. M-N).

Supplementary Table S6.

Abbreviations used for TCGA datasets.

Supplementary Table S7.

APE1-PPI bad prognostic signatures top regulators analysis. GeneXplain identification of the Top 3 putative master regulators of bad prognostic genes in the 11 selected TCGA cancer datasets (ranked by ascending Ranks sum). Bibliographic references are given for the association with the proliferation, apoptosis and resistance functional terms, indicating the involvement of top upstream regulators in these pathways (x indicates that no reference was found).

Supplementary Table S8.

GeneXplain identification of the Top10 putative master regulators of the APE1-PPI global network (ranked by ascending Ranks sum).

References

1. Antoniali, G., Marcuzzi, F., Casarano, E. & Tell, G. Cadmium treatment suppresses DNA polymerase δ catalytic subunit gene expression by acting on the p53 and Sp1 regulatory axis. *DNA Repair (Amst.)* **35**, 90–105 (2015).
2. Malfatti, M. C. *et al.* Abasic and oxidized ribonucleotides embedded in DNA are processed by human APE1 and not by RNase H2. *Nucleic Acids Res.* **45**, 11193–11212 (2017).
3. Antoniali, G. *et al.* Mammalian APE1 controls miRNA processing and its interactome is linked to cancer RNA metabolism. *Nat Commun* **8**, 797 (2017).
4. Rai, G. *et al.* Synthesis, biological evaluation, and structure-activity relationships of a novel class of apurinic/apyrimidinic endonuclease 1 inhibitors. *J Med Chem* **55**, 3101–12 (2012).

Supplementary Figure S1

a

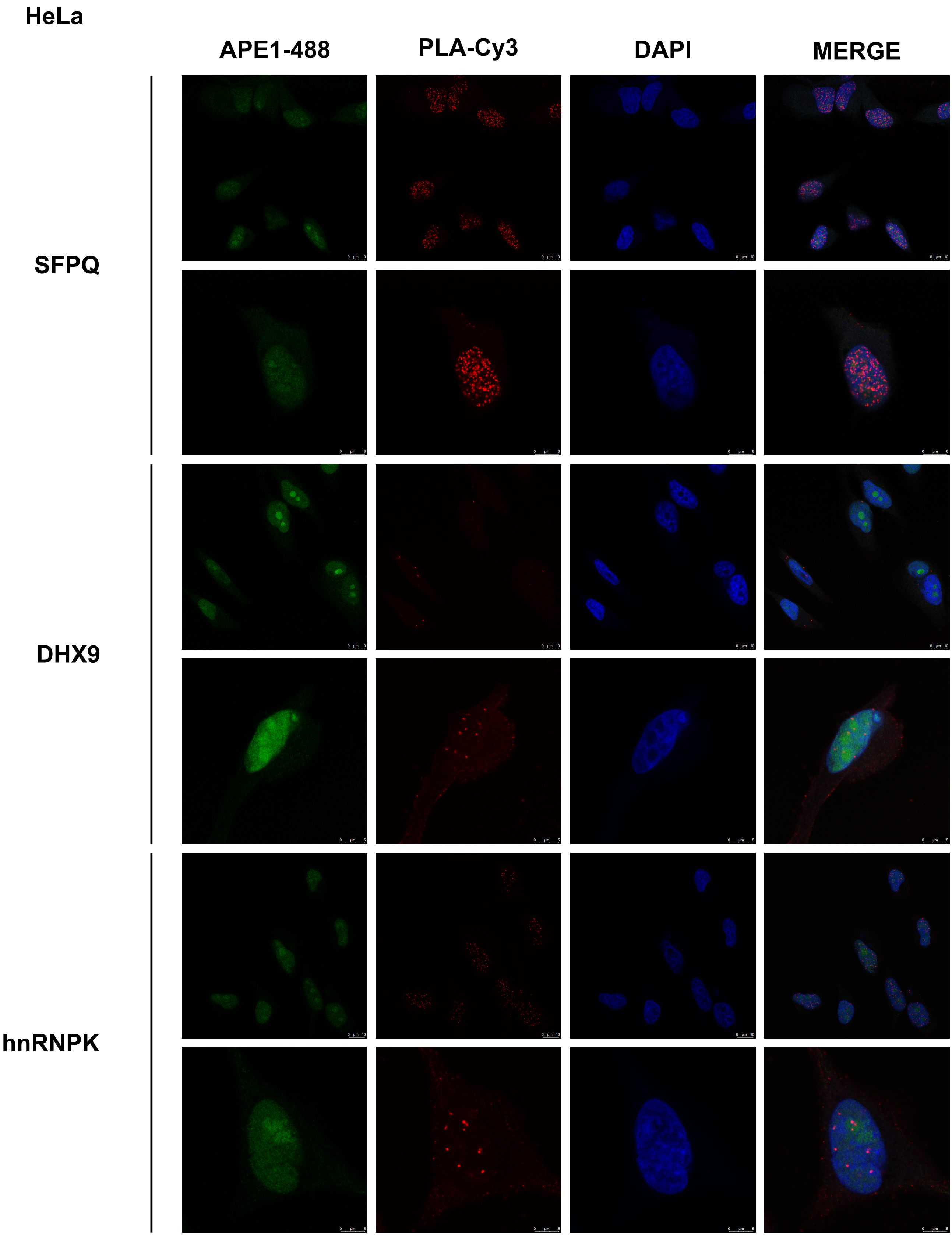

Supplementary Figure S1

b

A549
APE1-SFPQ

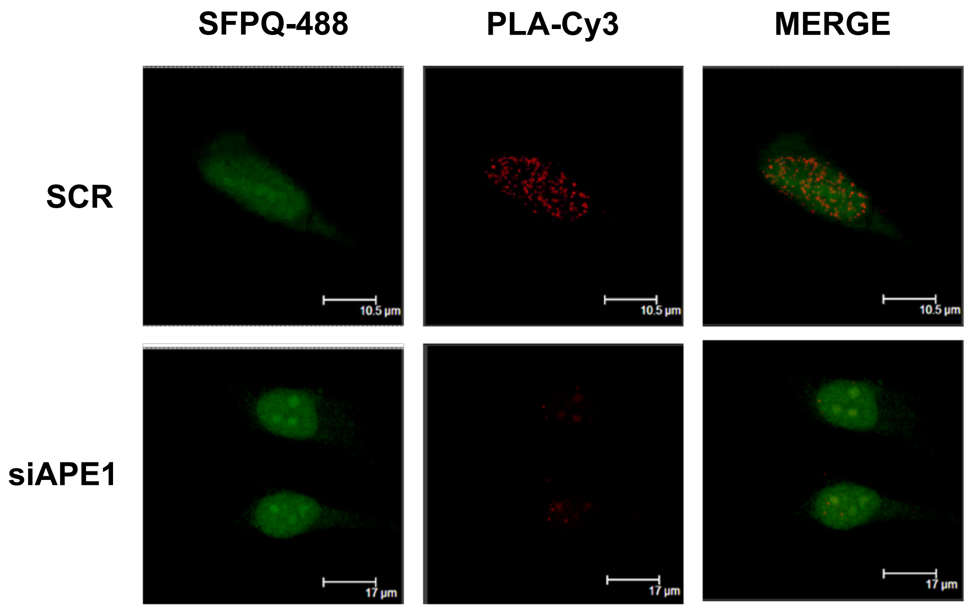

c

JHH-6
APE1-hnRNPA2B1

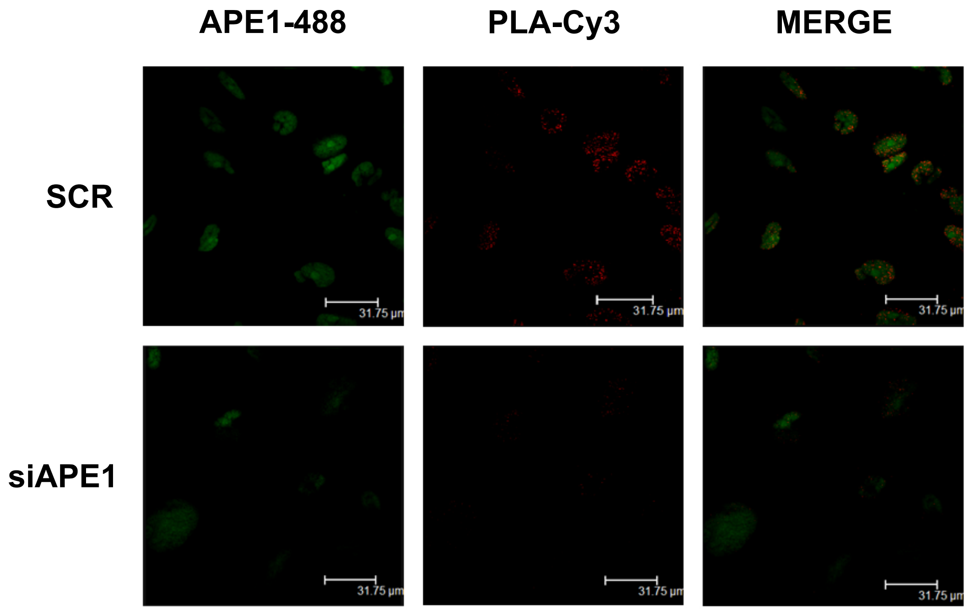

Supplementary Figure S2

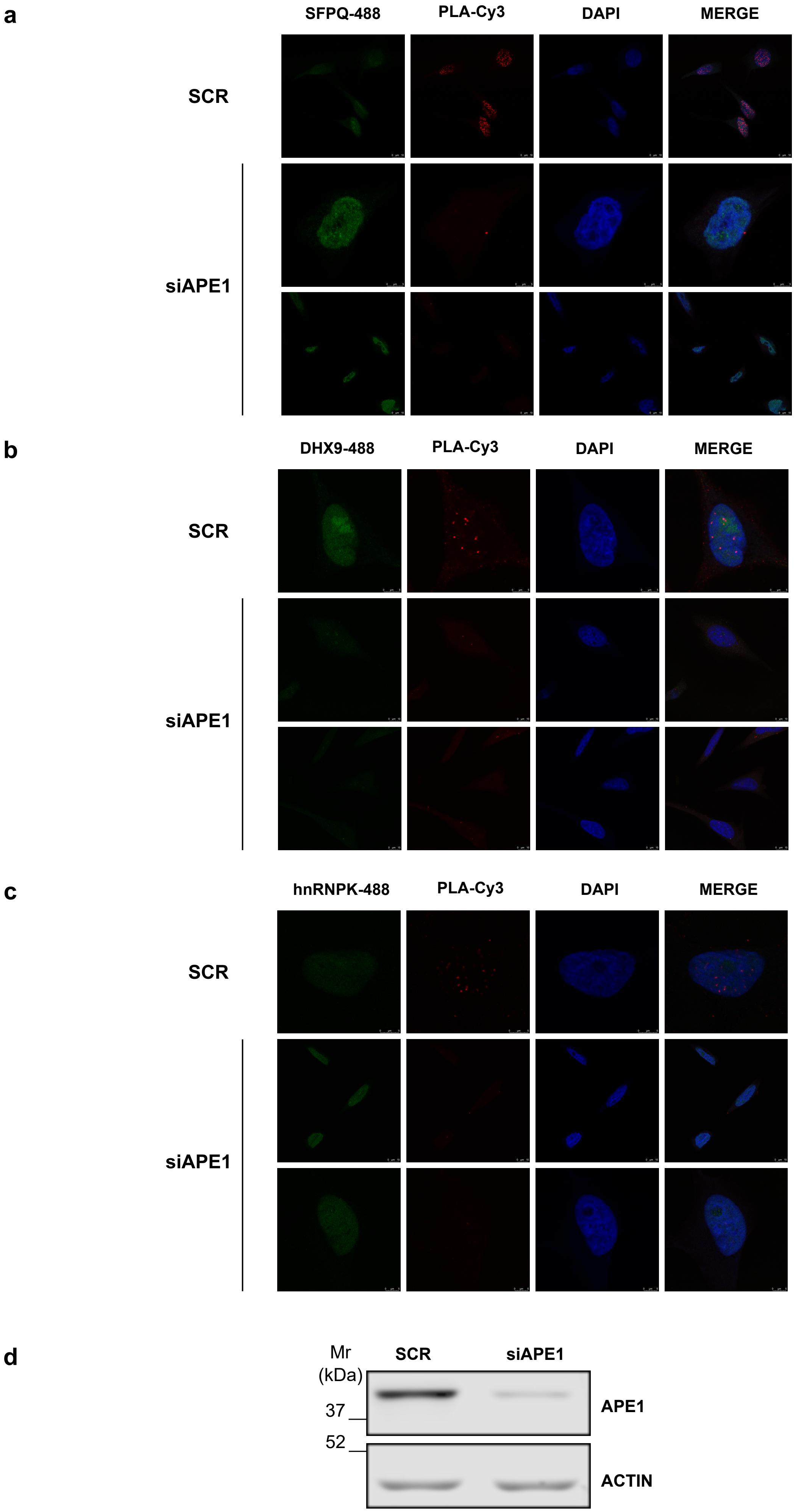

Supplementary Figure S3

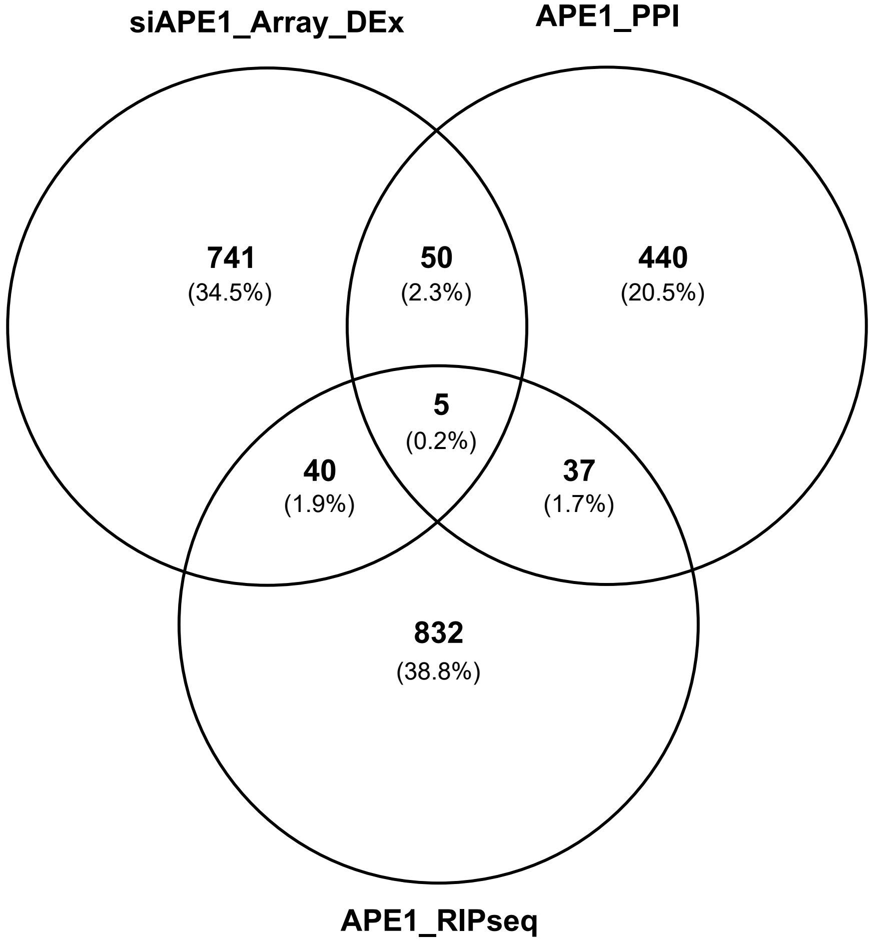

Supplementary Figure S4

a

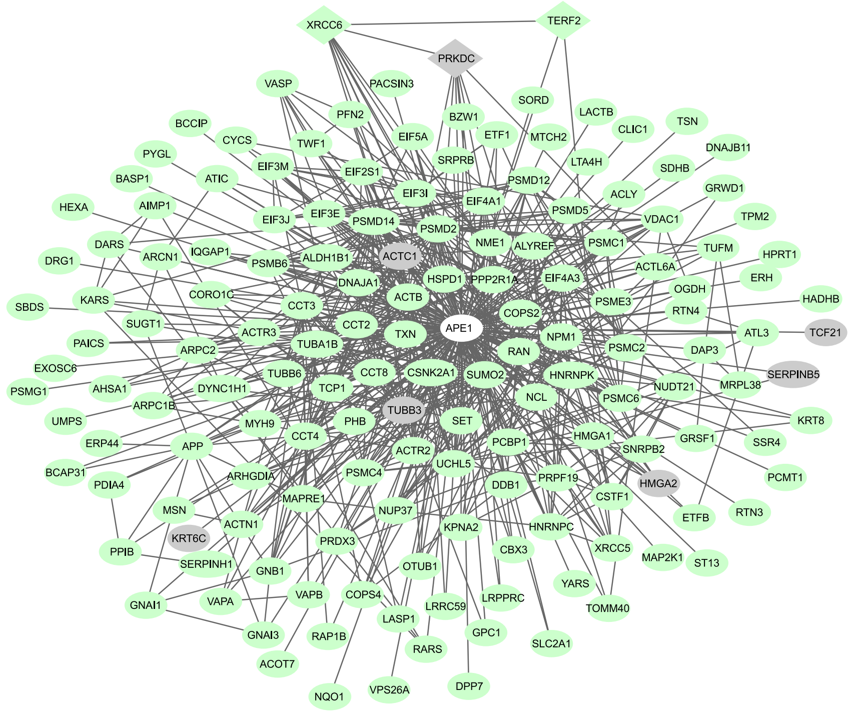

b

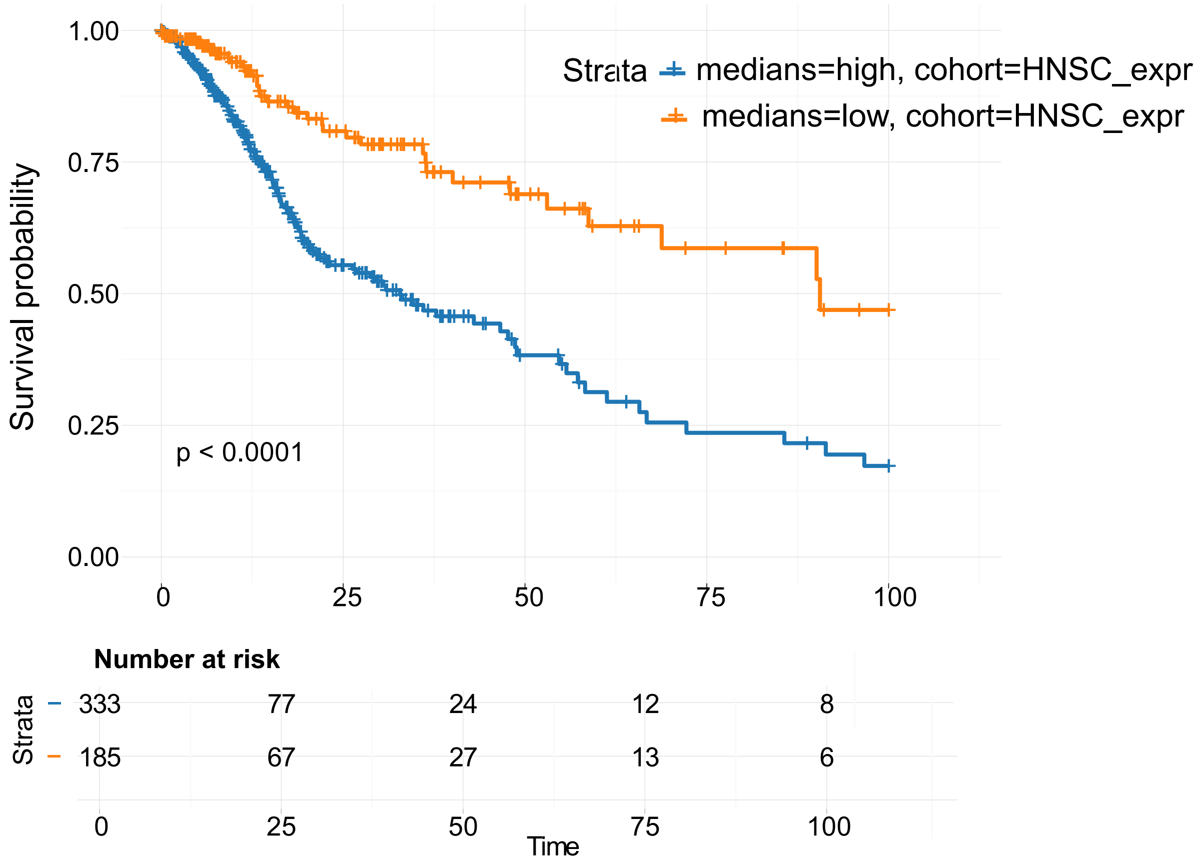

c

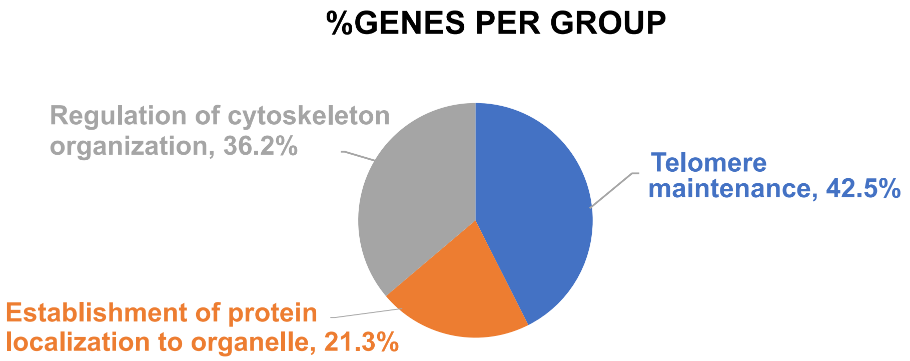

Supplementary Figure S5

a

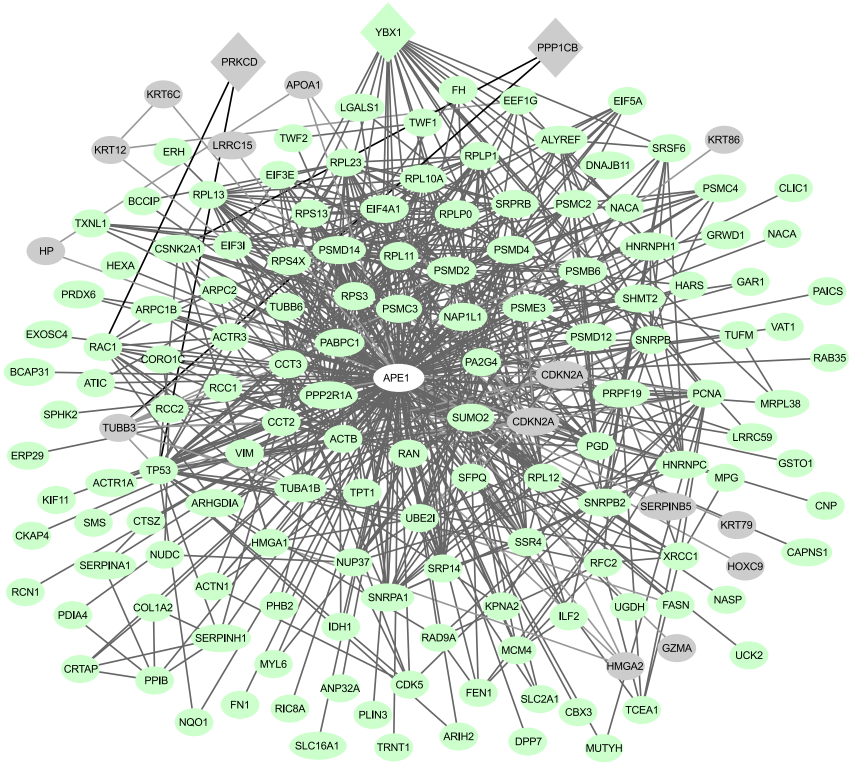

b

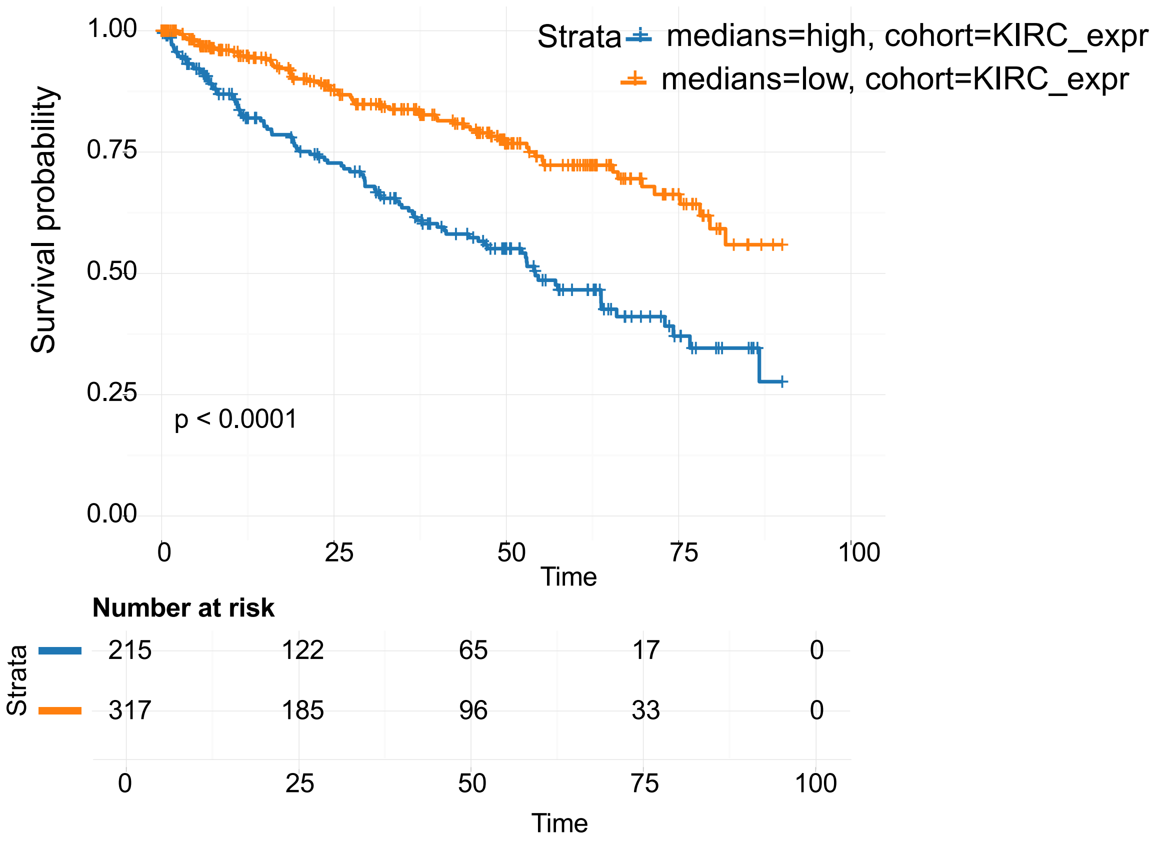

c

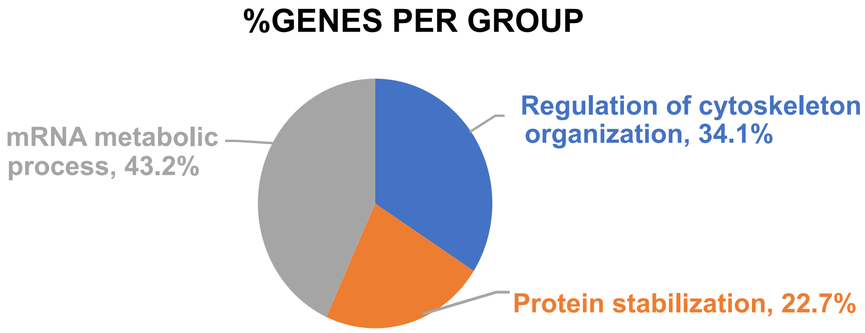

Supplementary Figure S6

a

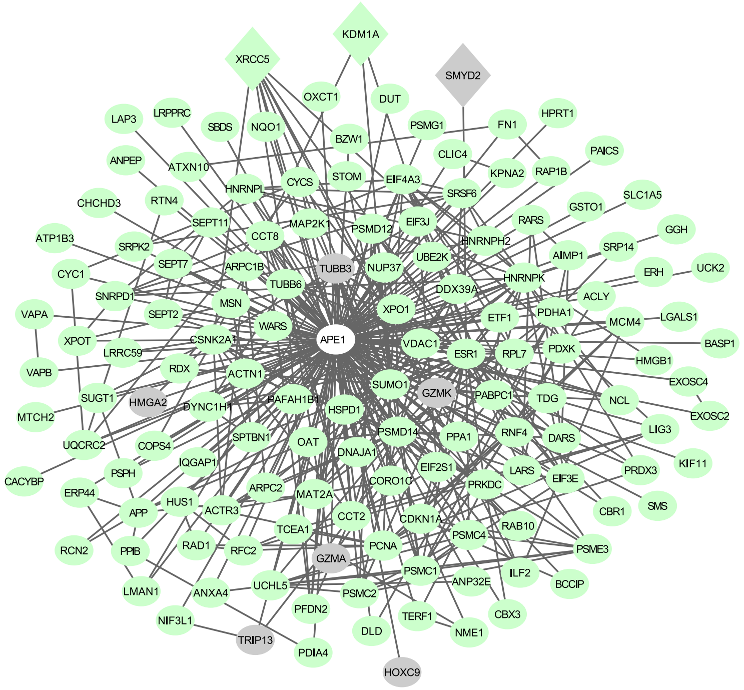

b

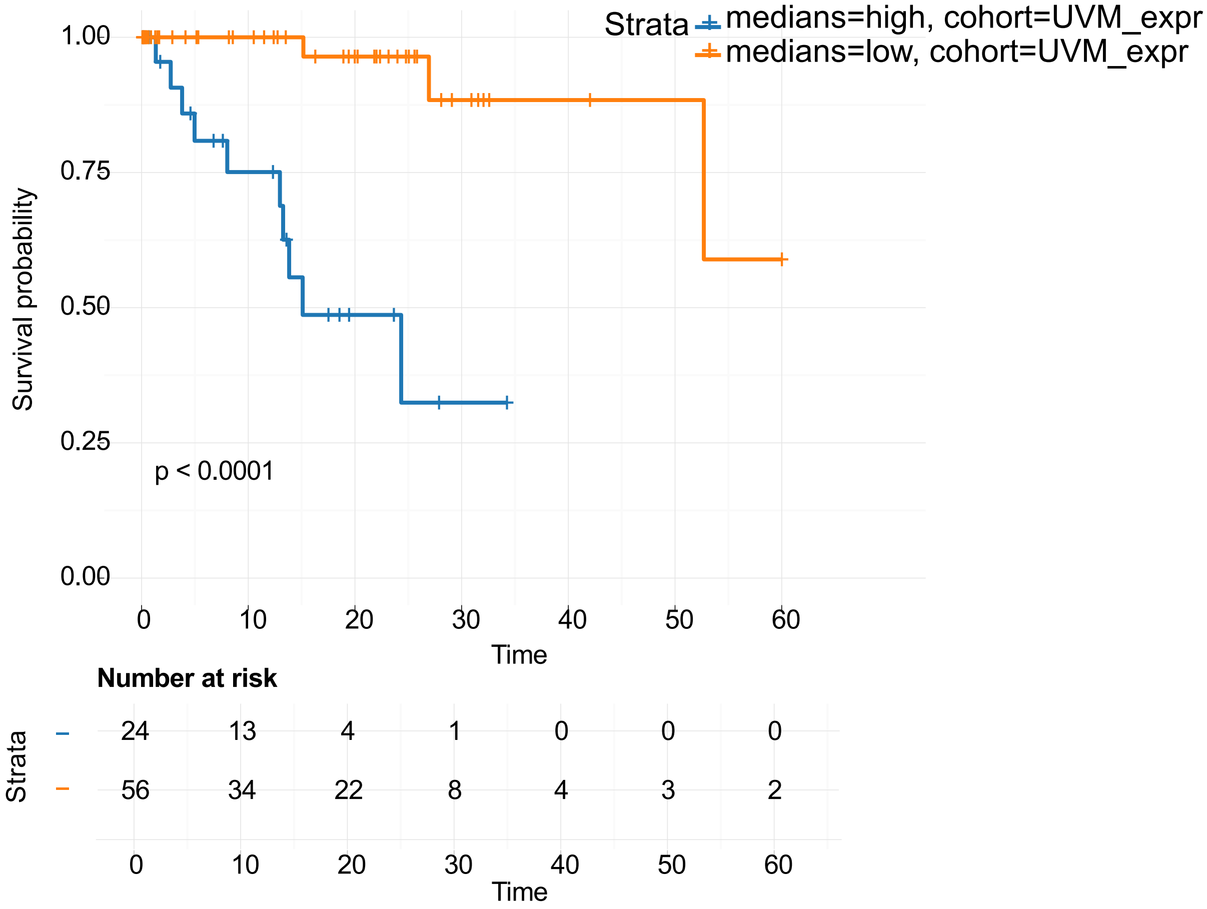

c

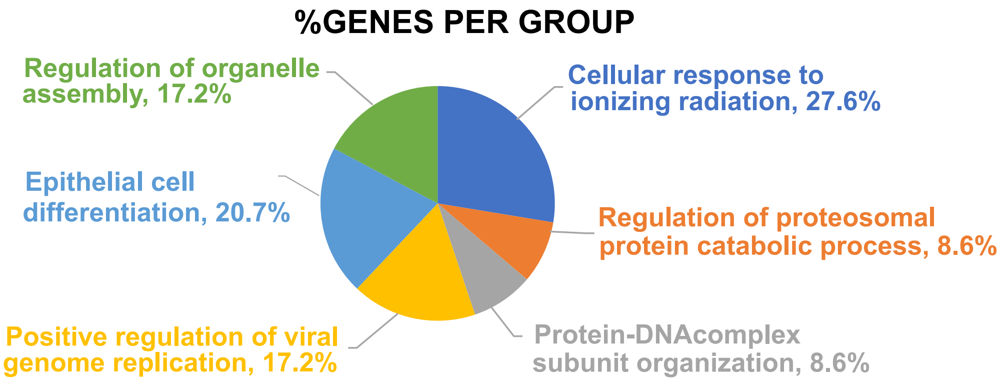

Supplementary Figure S7

a

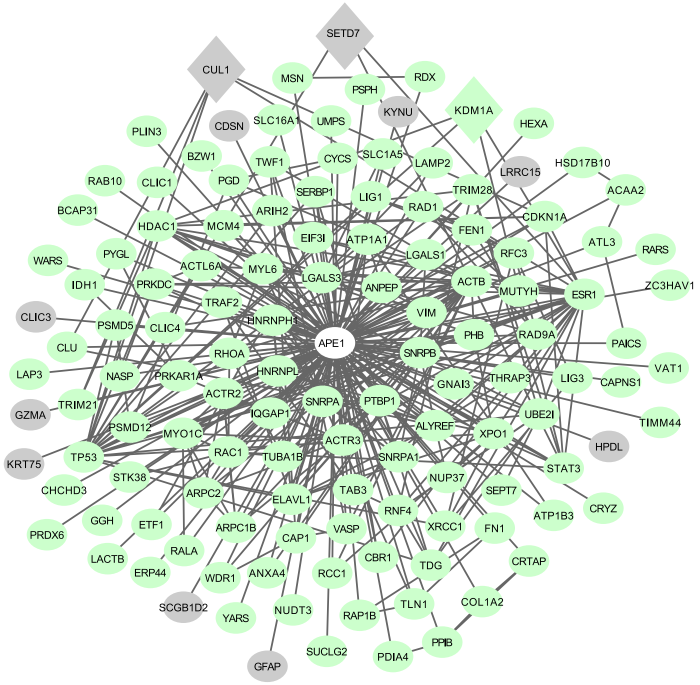

b

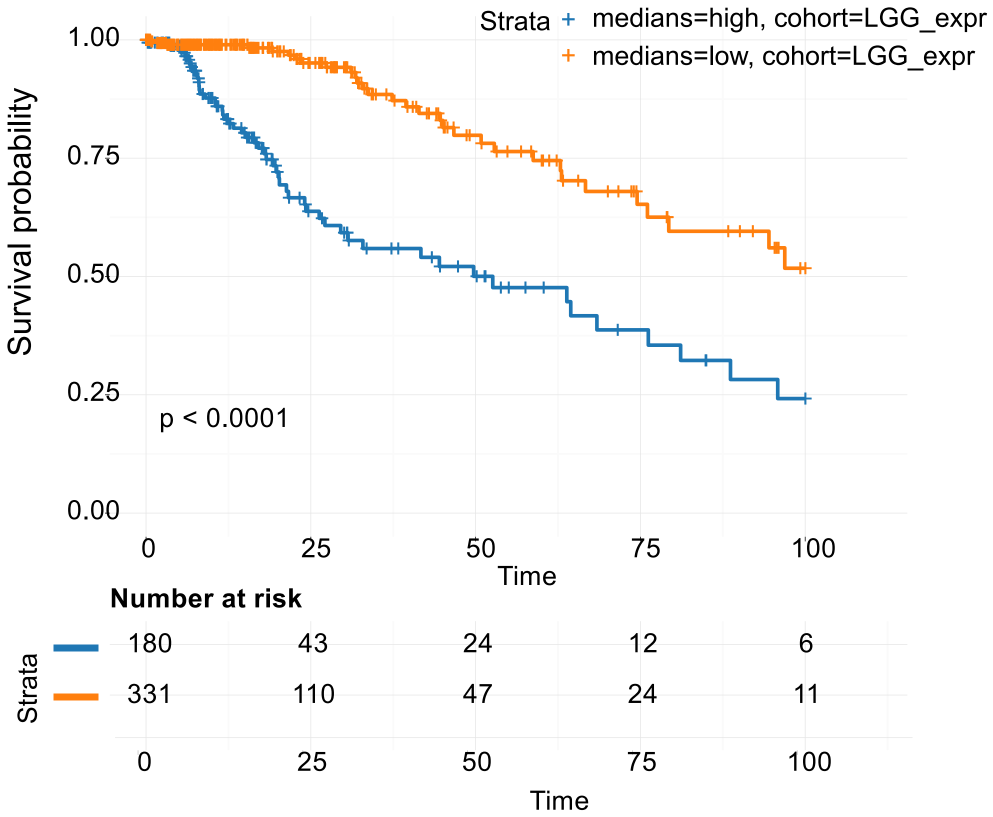

c

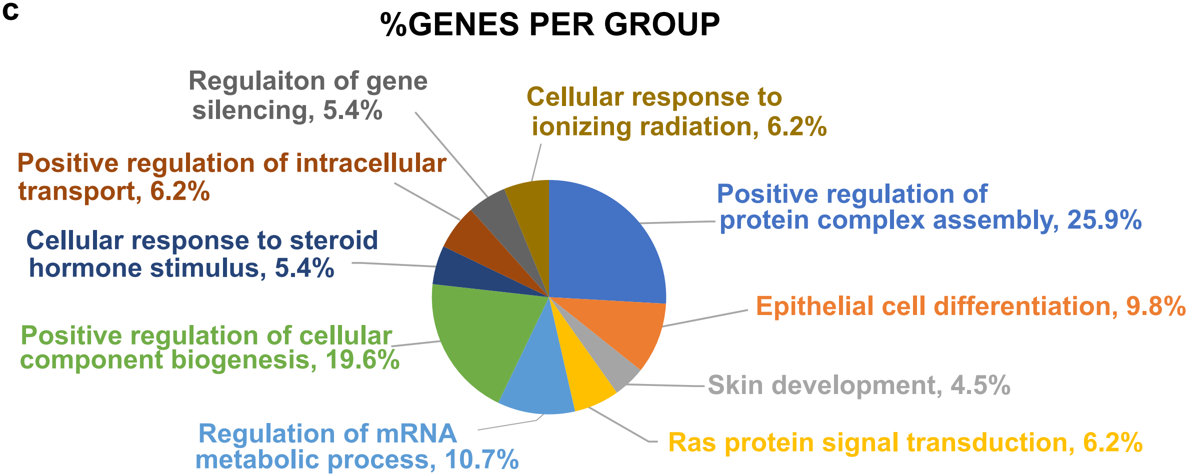

Supplementary Figure S8

a

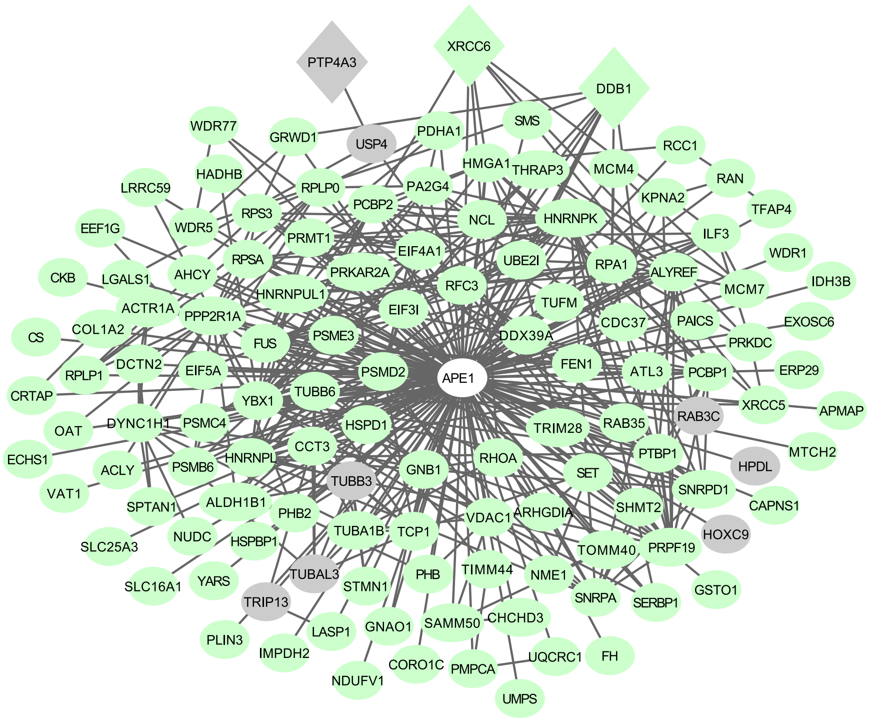

b

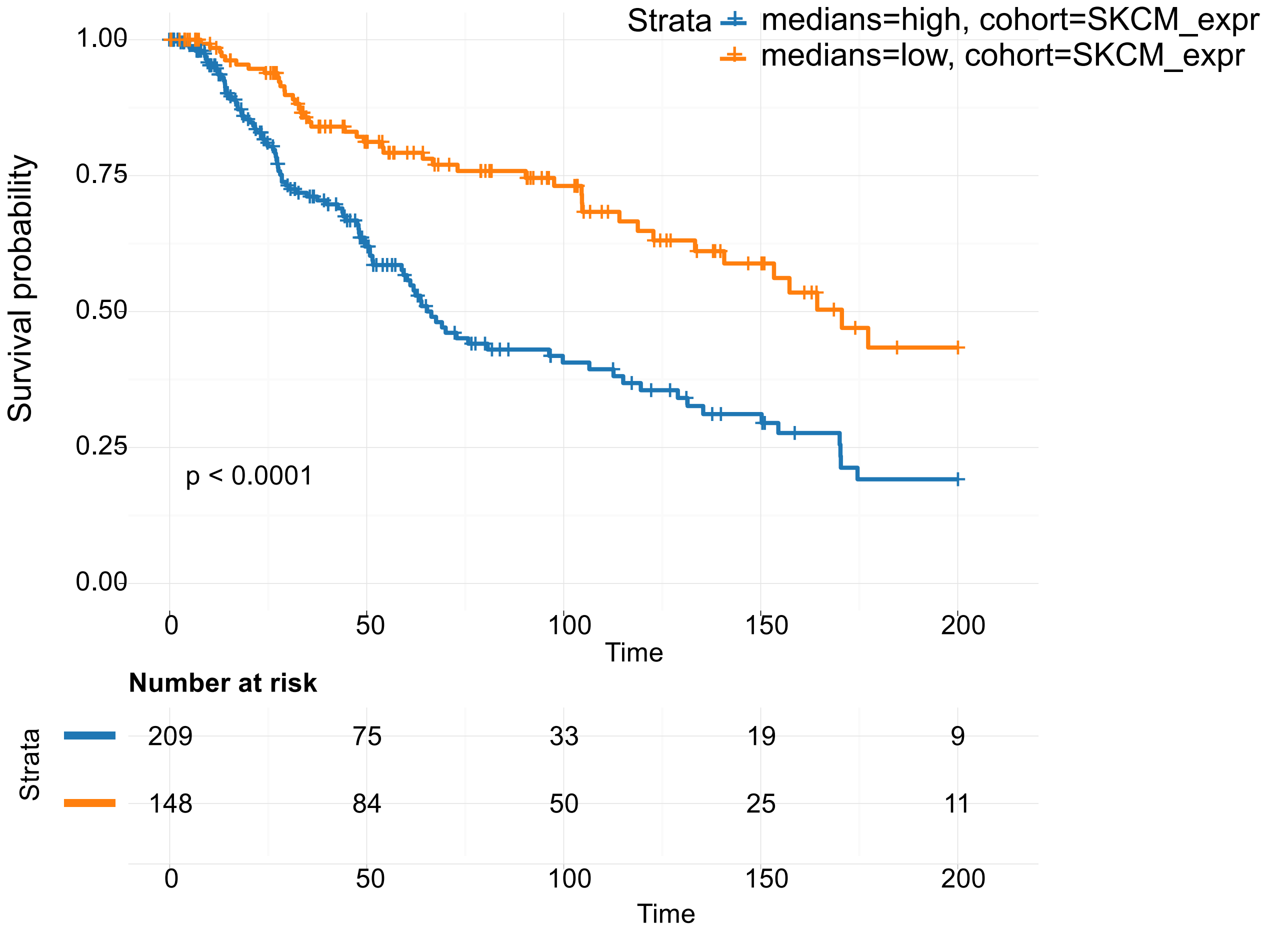

c

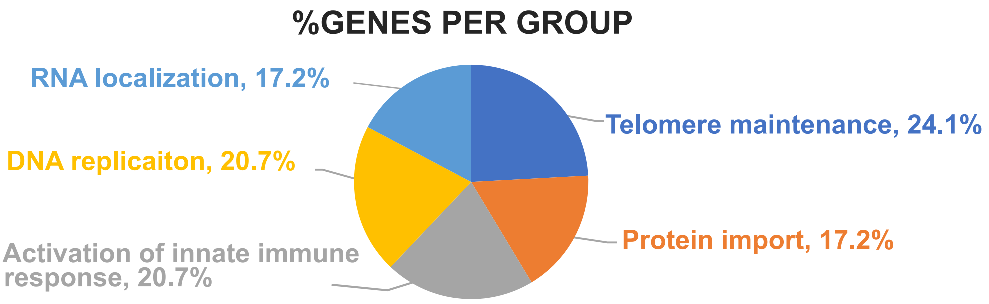

Supplementary Figure S9

a

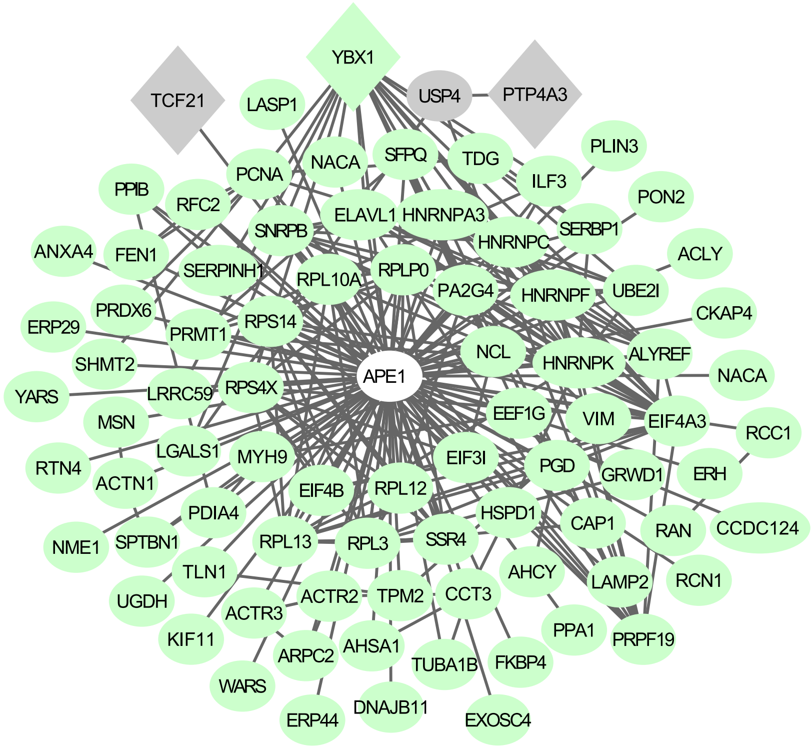

b

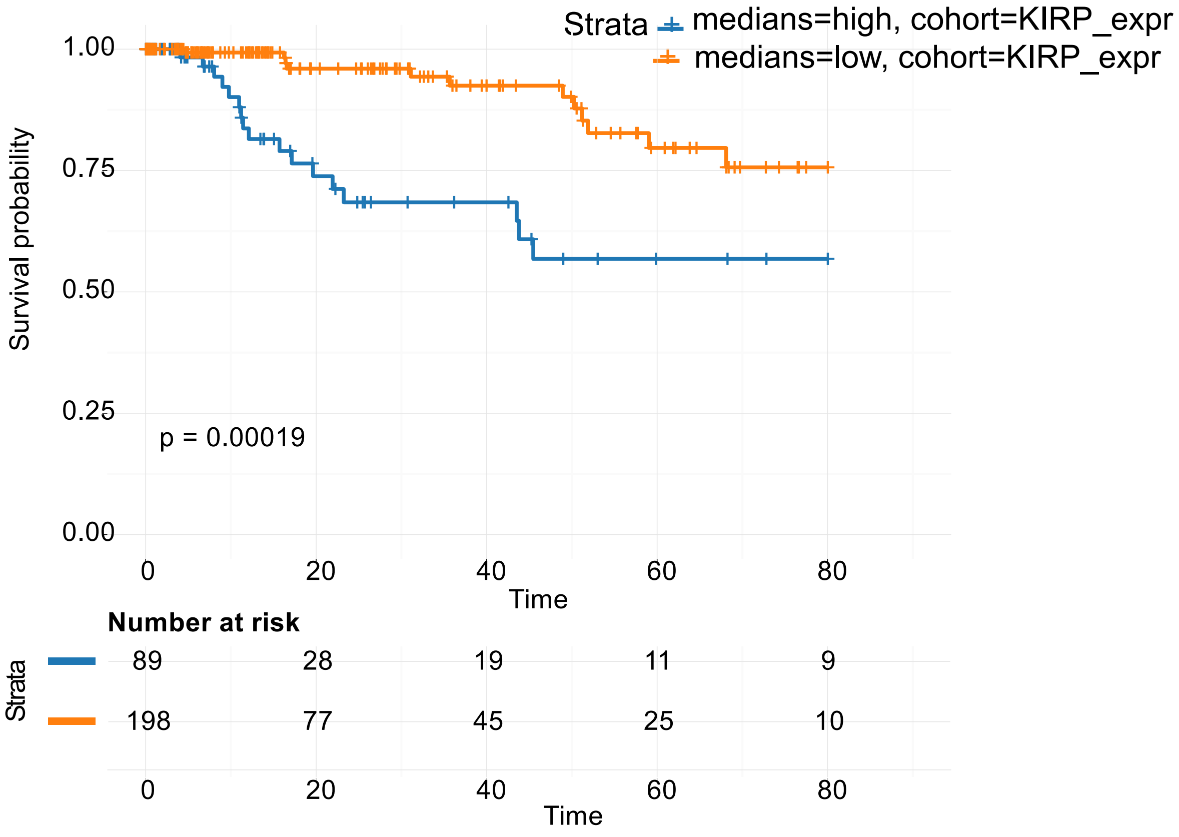

c

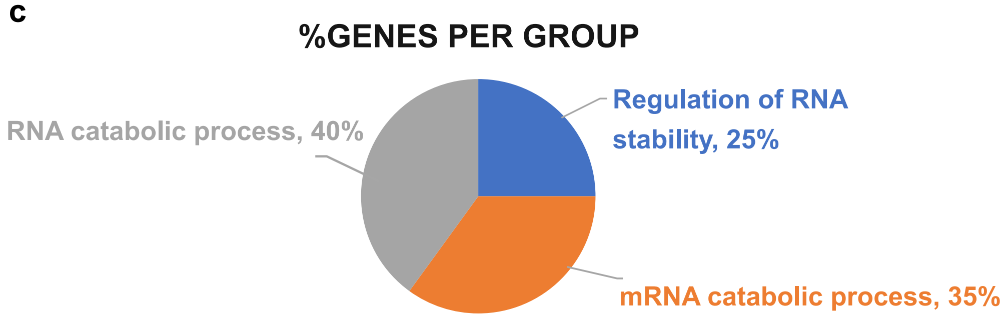

Supplementary Figure S10

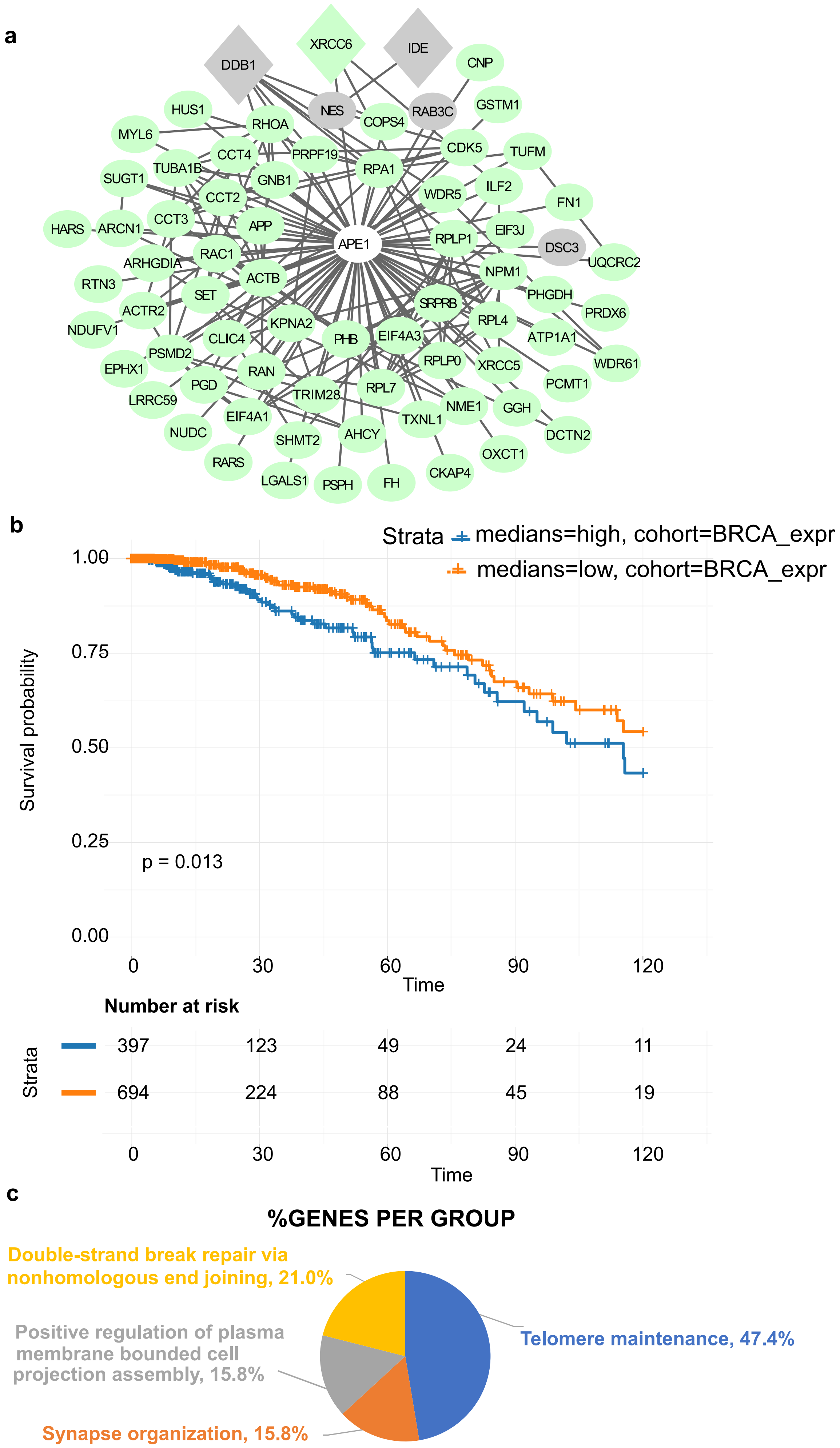

Supplementary Figure S11

a

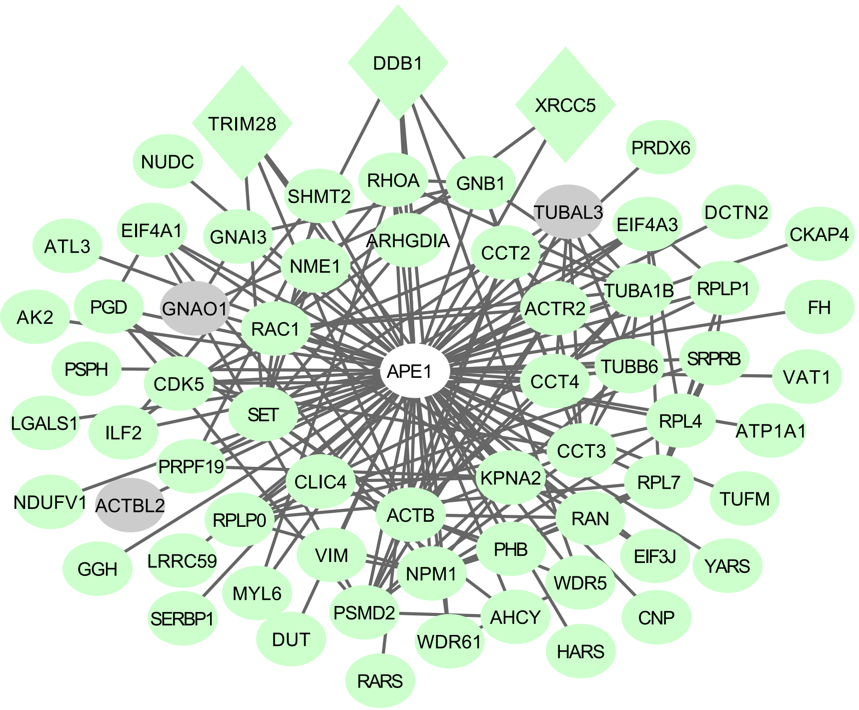

b

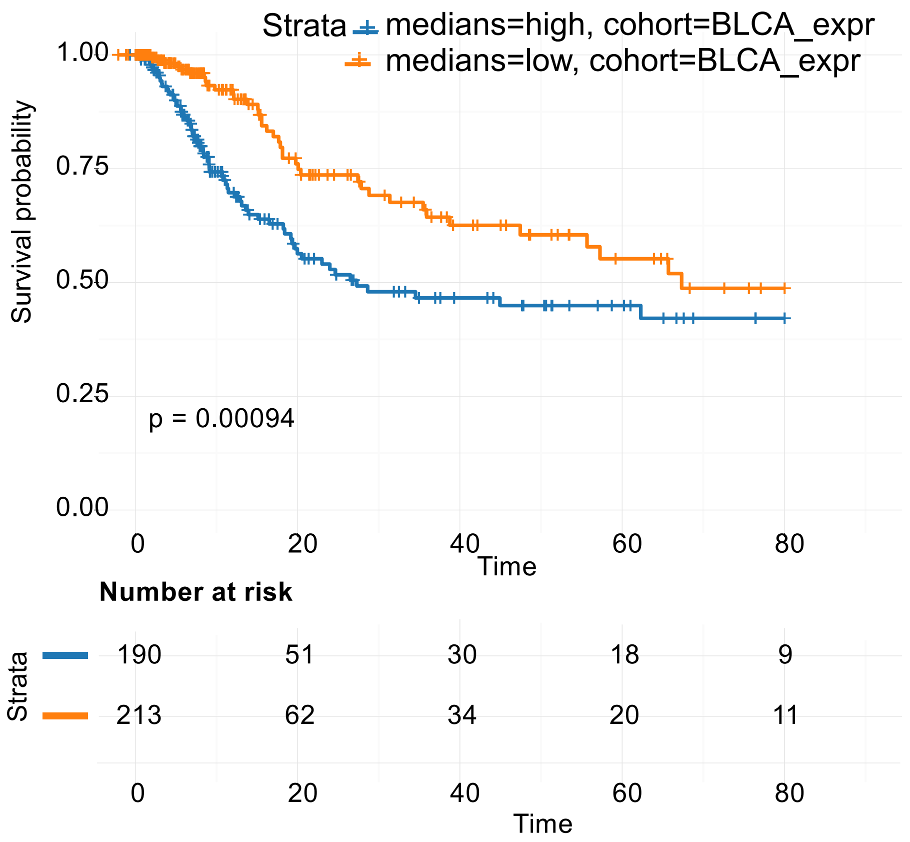

c

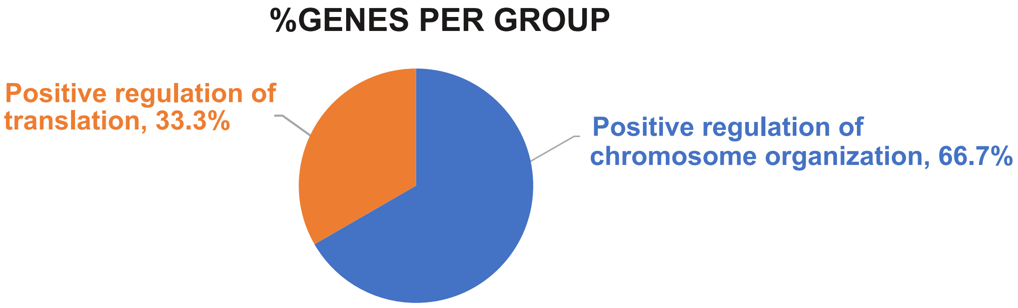

Supplementary Figure S12

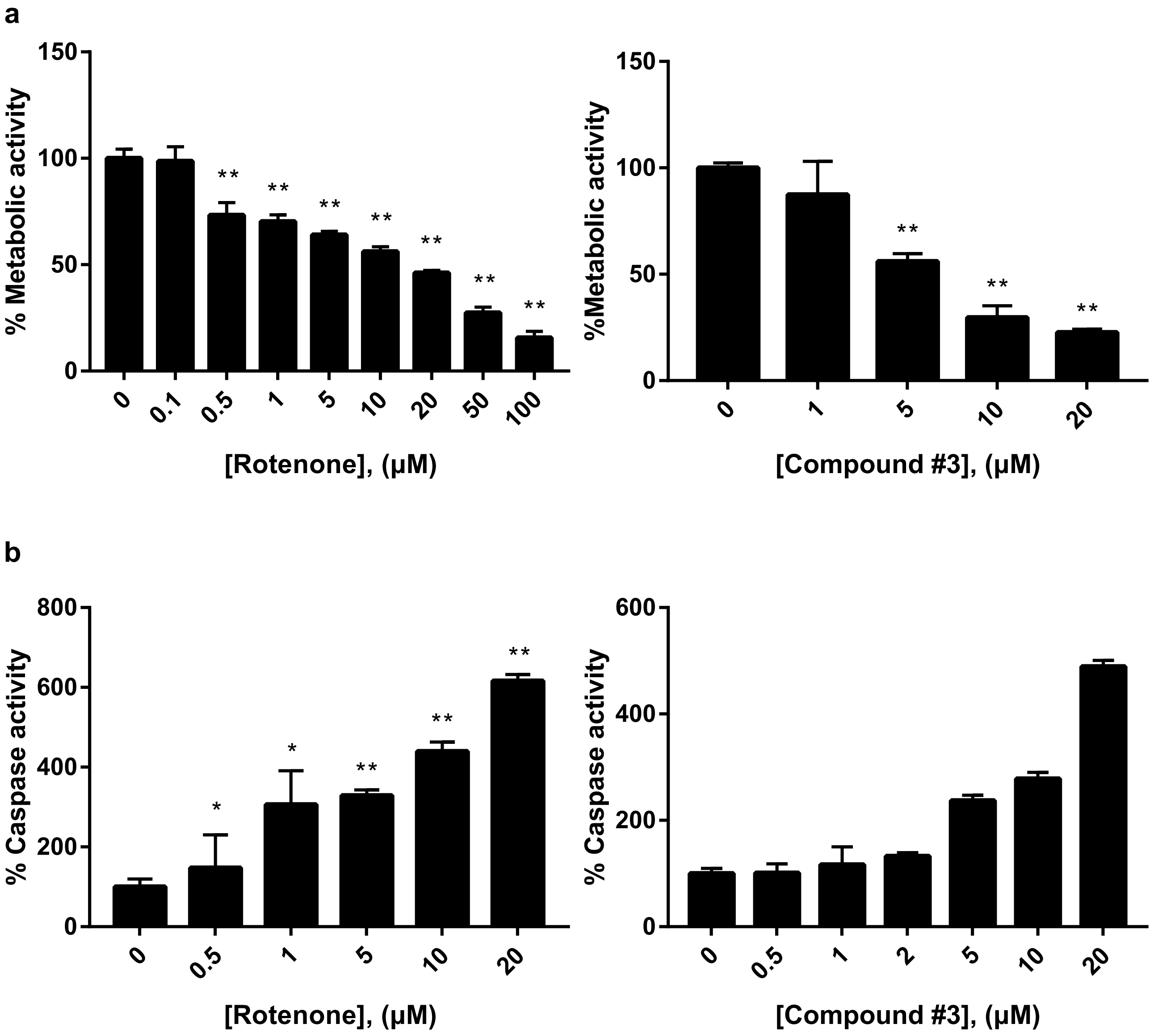

Supplementary Figure S13

a

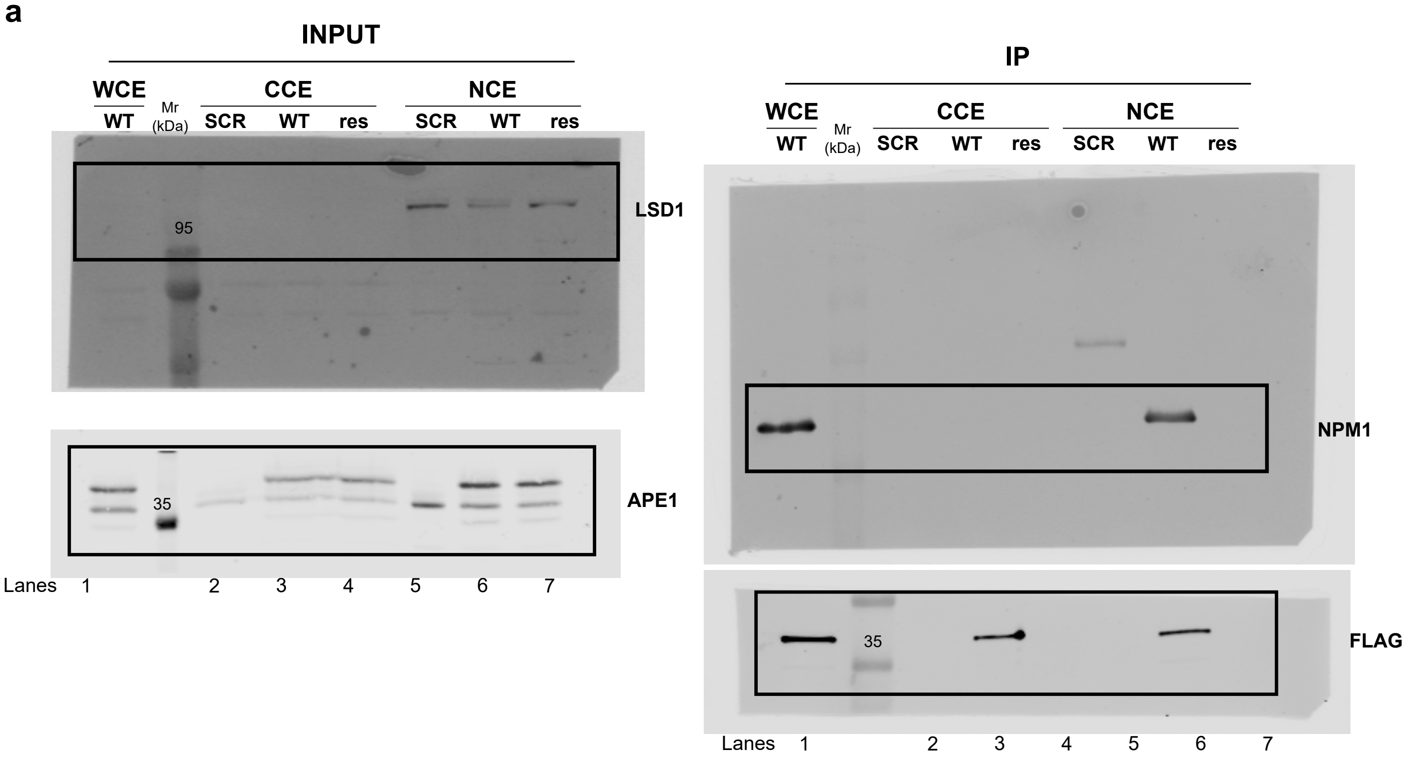

b

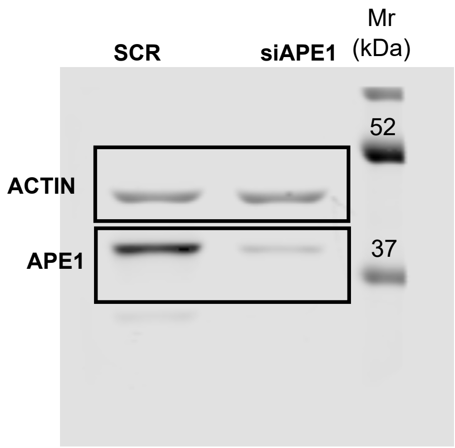

Supplement: Supplementary file 9 — Supplementary info. [file 41598_2019_56981_MOESM9_ESM.pdf]
